# Detecting haplotype-specific transcript variation in long reads with FLAIR2

**DOI:** 10.1186/s13059-024-03301-y

**Published:** 2024-07-02

**Authors:** Alison D. Tang, Colette Felton, Eva Hrabeta-Robinson, Roger Volden, Christopher Vollmers, Angela N. Brooks

**Affiliations:** Department of Biomolecular Engineering, University of California, Santa Cruz, USA

**Keywords:** FLAIR, ADAR, A-to-I editing, Long-read RNA-seq

## Abstract

**Background:**

RNA-seq has brought forth significant discoveries regarding aberrations in RNA processing, implicating these RNA variants in a variety of diseases. Aberrant splicing and single nucleotide variants (SNVs) in RNA have been demonstrated to alter transcript stability, localization, and function. In particular, the upregulation of ADAR, an enzyme that mediates adenosine-to-inosine editing, has been previously linked to an increase in the invasiveness of lung adenocarcinoma cells and associated with splicing regulation. Despite the functional importance of studying splicing and SNVs, the use of short-read RNA-seq has limited the community’s ability to interrogate both forms of RNA variation simultaneously.

**Results:**

We employ long-read sequencing technology to obtain full-length transcript sequences, elucidating cis-effects of variants on splicing changes at a single molecule level. We develop a computational workflow that augments FLAIR, a tool that calls isoform models expressed in long-read data, to integrate RNA variant calls with the associated isoforms that bear them. We generate nanopore data with high sequence accuracy from H1975 lung adenocarcinoma cells with and without knockdown of *ADAR*. We apply our workflow to identify key inosine isoform associations to help clarify the prominence of ADAR in tumorigenesis.

**Conclusions:**

Ultimately, we find that a long-read approach provides valuable insight toward characterizing the relationship between RNA variants and splicing patterns.

**Supplementary Information:**

The online version contains supplementary material available at 10.1186/s13059-024-03301-y.

## Introduction

Adenosine-to-inosine (A-to-I) editing is one of the most common forms of RNA editing in organisms with a developed central nervous system [1–5]. As inosines are recognized by cellular machinery as a guanosine, one potential downstream effect of A-to-I editing is the alteration of coding sequence. There are numerous cases of A-to-I recoding identified as essential for normal brain function [6–8] and yet other cases where recoding worsens disease prognosis [9–11]. In addition to recoding potential, inosines can affect RNA splicing in a *cis-*regulatory manner through the disruption of splice sites or splicing regulatory elements, leading to the creation of alternatively spliced mRNAs [12–14]. Considering that 95–100% of multi-exon genes are alternatively spliced [15], the effects of A-to-I editing on coding changes, regulatory elements, and alternative splicing require further study to elucidate.

The expression of ADAR1 is ubiquitous and the A-to-I editing that ADAR1 mediates on dsRNAs is widespread [16]. Previous literature has described the role of ADAR1 in autoimmune diseases [17–19], such as in the case of decreases in editing levels in particular dsRNAs resulting in MDA5-dependent interferon response and inflammation [20]. Additionally, aberrant ADAR activity has been linked to many other diseases [6, 7, 21–25], including diseases of the lung and blood, in which ADAR overexpression is associated with increased malignancy [10, 11]. In H1975 lung adenocarcinoma (ADC) cell lines, ADAR is not only upregulated but also has been shown to bind to and edit focal adhesion kinase (FAK), increasing both FAK expression and mesenchymal properties of the cells [10]. The connection of ADARs with diseases, in particular lung adenocarcinoma, in addition to the influence that ADARs have on the transcriptome underscores the importance of characterizing the complete RNA sequences that bear inosine edits.

Despite appreciable efforts to map A-to-I editing sites [4, 26], there is an absence of studies examining the full transcriptional context of inosines. Previous efforts to document A-to-I editing using short-read sequencing report the genomic position of edited sites [4, 26, 27] and our goal is to analyze the transcripts where edits reside. To investigate the transcriptome-wide impact of ADAR in lung ADC, we performed nanopore long-read cDNA sequencing of H1975 lung ADC cells with ADAR knockdown. We overcame the relatively high error rate of nanopore sequencing by using the Rolling Circle Amplification to Concatemeric Consensus (R2C2) nanopore cDNA sequencing method [28]. R2C2 greatly lowers the error rate of nanopore cDNA sequencing through the increase of single molecule coverage, yielding a median 98.7% base accuracy [29]. Accurate, long reads allow us to resolve full-length transcripts and RNA editing, equipping us to better understand the role of ADAR editing in the cancer transcriptome.

In RNA-seq data, there is ambiguity as to whether mismatches to the reference genome correspond to (1) somatic or germline variants; (2) RNA edits in which one nucleotide is edited to read as another, or, in the case of nanopore direct RNA sequencing; and (3) modified RNA nucleotides. Although R2C2 is unable to preserve RNA modifications, we have devised a tool to phase and associate consistent mismatches to isoform models given long reads, agnostic to the kind of alteration that accounts for the mismatch. We refer to these mismatch-aware isoforms generally as haplotype-specific transcripts (HSTs), with a set of variants occurring on the same transcripts designated a “haplotype.” In efforts to jointly identify isoform structure and the potentially stochastic nature of inosine positions in nanopore data, we introduce a computational software for identifying HSTs. We built upon the isoform detection tool FLAIR [30], which is one among many tools (Stringtie2 [31], FLAMES [32], TALON [33], MandalorION [34]) developed for this purpose. FLAIR was initially developed to identify transcript models in long reads to hone in on subtle splice site changes; the original FLAIR method was primarily concerned with error-prone and truncated reads, with minimal consideration for sequence variation. This variant-aware FLAIR, called FLAIR2 [35], incorporates mismatches from a variant caller into transcript models for an arbitrary number of haplotypes as would be useful for grouping editing events, distinguishing itself from other allele-specific expression tools for long reads such as LORALS [36], IDP-ASE [37], and FLAMES [32].

Here, we use FLAIR2 to detect haplotype-specific transcripts in a diploid mouse hybrid long- and short-read dataset and compare changes in inosine editing in the context of lung cancer. We sequenced lung ADC cell lines with and without *ADAR1* knockdown using Illumina RNA-seq as well as R2C2 nanopore sequencing. Paired with the development of the necessary computational framework for full-length isoform and RNA editing analyses, we reveal new insights into long-range A-to-I edits and demonstrate the power of long-read sequencing as a tool for the transcriptome-wide identification of inosines.

## Results

### FLAIR2 is a variant-aware isoform detection pipeline

In an effort to build user-friendly computational workflows for nanopore data, we previously developed a tool called Full-Length Alternative Isoform analysis of RNA (FLAIR). FLAIR calls isoform structures and performs various isoform-level analyses of nanopore cDNA [30] and direct RNA sequencing data [38]. We designed the FLAIR workflow to account for the increased error rate of long reads, in particular for nanopore data. Previous work with FLAIR emphasized the discovery of isoform models and their comparison between sample conditions. We have adjusted FLAIR to incorporate phased variant calls to investigate haplotype-specific transcript expression in nanopore data. We also sought to improve FLAIR’s performance on isoform structure (transcript start and ends and exon-exon connectivity) by increasing sensitivity to annotated transcript isoforms.

The modified FLAIR workflow (FLAIR2) now begins with an alignment of all reads to the annotated transcriptome. The addition of this ungapped alignment step was designed to improve small or microexon detection for error-containing, spliced reads which are difficult to align to the genome [39]. Reads are assigned to an annotated transcript if they have high sequence identity with the transcript, with an emphasis on accuracy proximal to splice sites (see “Methods”). The annotated transcripts that have sufficient long-read support are included as part of the set of FLAIR isoforms. The remaining reads that are not able to be assigned to an annotated transcript are then used to detect novel transcript models (see “Methods”). The final, sample-specific isoform assembly includes the supported, annotated isoform models combined with the novel models. FLAIR is also capable of downstream analyses such as isoform quantification and differential expression tests of nanopore data, as described previously [30].

To compare the performance of FLAIR2 with our previous version of FLAIR, we investigated the transcript-level precision and sensitivity using previously published nanopore 1D cDNA Spike-in RNA Variant (SIRV) sequences [30], which represent a ground-truth for expected sequenced transcripts. Additionally, we compared FLAIR2’s performance against other more recently published tools, Stringtie2 [31] and FLAMES [32]. Demonstrating the enhanced performance of FLAIR2’s approach, it had marked improvement (37-point increase in transcript-level precision) over the previously published FLAIR, performed comparatively best in precision, and performed similarly to other tools in terms of sensitivity (Table S1). One example of improvements expected in FLAIR2 include cases where genomic alignments are less accurate than alignments to an annotated transcript, such as in cases where the updated FLAIR2 is now capable of distinguishing between an annotated small intron and a deletion (Fig. S1).

A more comprehensive evaluation of FLAIR2 has been performed through the Long-read RNA-seq Genome Annotation Assessment Project (LRGASP) Consortium [40]. This systematic and community-organized evaluation compared the performance of FLAIR2 using different library preparation methods and sequencing platforms: cDNA sequencing with Oxford Nanopore Technologies (ONT) or Pacific Biosciences (PacBio) and direct RNA sequencing with ONT. Additionally, there were multiple sample types including real data from human and mouse, spike-in variants, and simulated data. Finally, there were multiple benchmarks with and without a ground truth. FLAIR2 was found to be one of the top performing tools for the detection of annotated and novel transcripts using multiple benchmarks for ONT and PacBio reads, as well as top-performing for quantification [40]. However, an evaluation of FLAIR2 performance of R2C2-ONT sequencing was not included in the LRGASP assessment.

To evaluate FLAIR2 with nanopore R2C2 sequencing, we utilized the publicly available SIRV-set4 R2C2 sequencing data from the LRGASP consortium along with the publicly available performance results of the tools Bambu [41], IsoQuant [42], Lyric [43], Mandalorion [34], and TALON [33]. This set of SIRVs contains “spliced SIRVs” which are synthetic transcripts with alternative splicing patterns to mimic human gene RNA processing complexity. Also included in sequencing were “unspliced SIRVs,” a set of synthetic transcripts with no shared alternative splicing patterns that were much longer transcripts (4–12 kb). On the LRGASP R2C2 spliced SIRV data, FLAIR was among the top performing tools in both sensitivity and precision; however, on the long, unspliced SIRVs, other tools such as Bambu and IsoQuant had superior sensitivity and precision (Table S2). As noted in the LRGASP assessment [40], most library preparation methods produced a majority of sequences less than 4 kb; therefore, few reads fully sequenced a long SIRV from end-to-end [40]. Even in the annotation-reliant mode, FLAIR will only determine if an annotated isoform is present if there is read-level evidence supporting the entire isoform, while other tools may accept partial support and weight already annotated isoforms more heavily when calling isoforms. Reporting only the annotated transcripts with high-confident, full-read support is a decision that allows FLAIR more confidence in novel isoform detection, at the expense of low sensitivity on longer transcripts with partial support. Additionally, we assessed FLAIR2 using the WTC-11 R2C2 data from LRGASP with benchmarks using orthogonal data support and a manual annotation performed by GENCODE [44]. FLAIR is the only tool that had the top 3 performance using all metrics including the percentage of annotated transcripts with full orthogonal support (%SRTM: 5′ end CAGE-seq, 3′ end Quant-seq, and short-read splice junction support) and percentage of novel transcripts with full orthogonal support (%SNTM) (Table S2). Using the GENCODE manual annotation as a benchmark, all tools had a weaker performance for novel transcript detection; however, FLAIR had the best sensitivity and 2nd best precision for detecting novel transcripts (Table S2). Overall, FLAIR2 has improved its transcript detection approach over the previous version and is one of the top performing tools for both annotated and novel transcript isoform detection using a variety of library preparation methods and sequencing approaches.

### Assessing FLAIR2 for haplotype-specific transcript detection

In addition to improvements for isoform detection, we developed FLAIR2 to be able to report isoforms along with their associated haplotype using provided variant calls. To integrate sequence variants into transcripts detected with FLAIR, we applied both longshot [45] and PEPPER-Margin-DeepVariant [46] to call variants in long-read data independent of the isoform calling. Both variant callers were developed for diploid variant calling and phasing in long reads. Following isoform identification, FLAIR2 has two modalities for phasing variants to discover variant-aware transcript models. The first uses phasing information from longshot, which is comprised of a phase set determined for each read as well as a set of variants corresponding to each phase set. FLAIR2 checks whether multiple reads that are assigned to the same isoform are also assigned by longshot to the same phase set. If these conditions are met with sufficient support for an isoform and phase set, then all variants belonging to that phase set will be associated with that isoform.

**Fig. 1:**
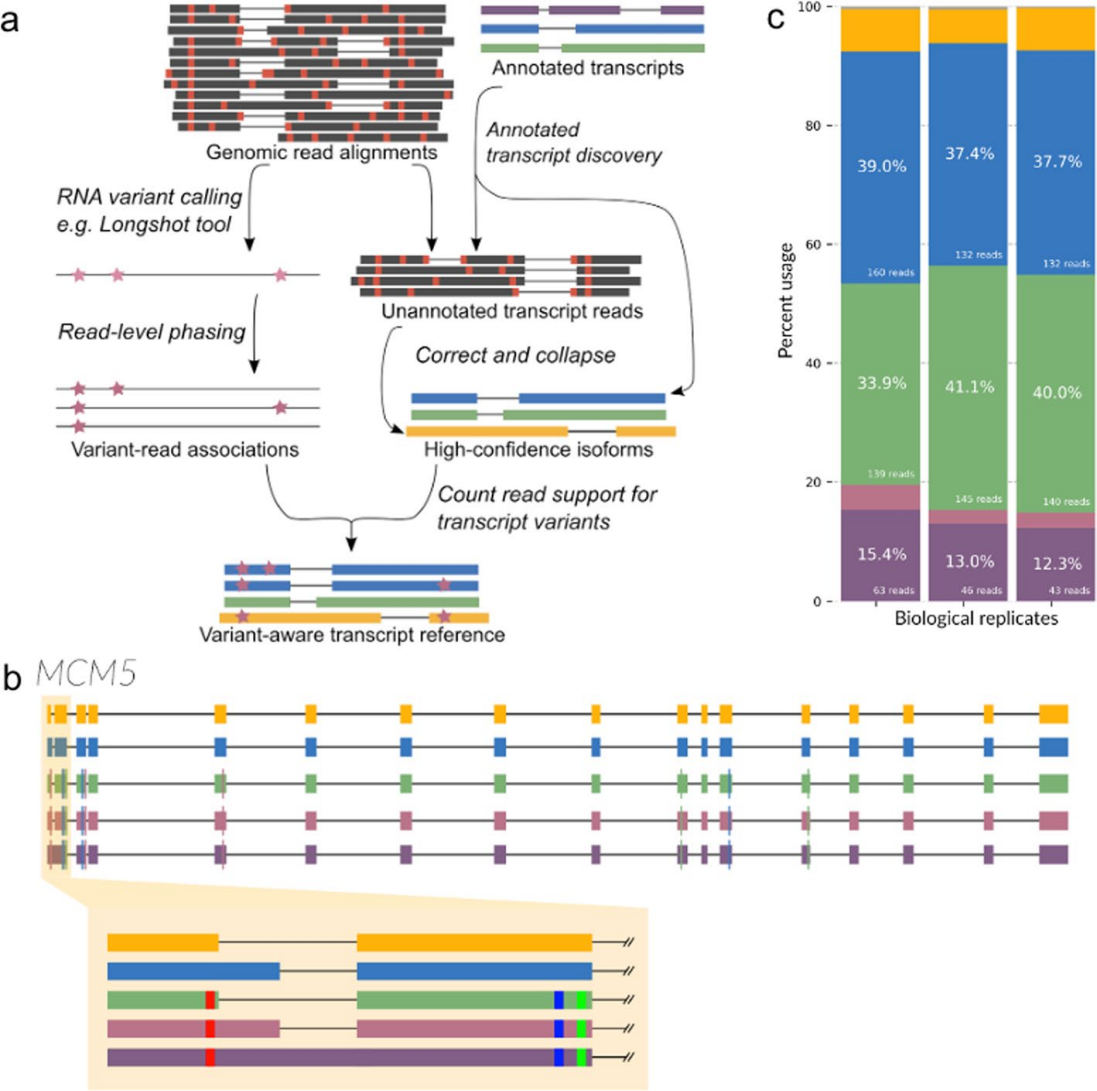
Variant-aware transcript detection by FLAIR2 identifies haplotype-specific transcript isoform bias. **a** Full FLAIR2 computational workflow for identifying haplotype-specific transcripts in long reads. For annotated transcript discovery, long reads are aligned to annotated transcript sequences and inspected for their overall match and read support at annotated splice junctions and transcript ends. The genomic alignments for reads that are not assigned to an annotated transcript are corrected and collapsed for unannotated isoform discovery. User-provided unphased/phased RNA variant calls can be associated with reads using FLAIR2; last, FLAIR2 counts the number of variant sets comprised by the reads assigned to each transcript model to determine variant-aware transcripts. Red ticks indicate mismatches; purple stars indicate RNA variants. **b** FLAIR transcript models for Mcm5 with the highest expression are plotted using different colors for each transcript’s exons. The highlighted portion shows alternative splicing and the smaller blocks within exons indicate variants. **c** Stacked bar chart showing the proportion of transcript expression of transcripts from b as matched by color for each of the replicates sequenced

For the second modality of variant-aware isoform detection, since we anticipated working with RNA edits and potential cancer-related aneuploidies that may result in more than two consistent haplotypes, FLAIR2 can approach phasing variants in a manner that is agnostic to ploidy: (1) from the isoform-defining collapse step, FLAIR2 generates a set of reads assigned to each isoform; (2) given variant calls, FLAIR2 tabulates the most frequent combinations of variants present in each isoform from its supporting read sequences; so (3) isoforms that have sufficient read support for a particular haplotype or consistent collection of variants are determined (Fig. 1a). This latter method of phasing focuses solely on the frequency of groups of mismatches that co-occur within reads and does not use ploidy information to refine haplotypes, allowing for the generation of multiple haplotypes within a gene and transcript model. This approach to phasing relies on reads with higher accuracy such as R2C2, and is not as robust to reads with higher error rates as it may create erroneous collections of variants. We provide an example of complex multiple haplotype calling where, given variant calls with simulated nanopore data with 99% accuracy and sufficient coverage of each haplotype, FLAIR2 incorporates 15/15 variants correctly (Fig. S2).

We tested the FLAIR2 isoform discovery pipeline on R2C2 data generated from Castaneus x Mouse 129 hybrid mouse embryonic stem cells [40] where we expect evidence of HSTs partitioned by known parental haplotypes [44]. Integrating longshot’s phased diploid variant calls, we identified transcripts that were significantly associated with one haplotype compared to other transcripts in the gene (Fisher’s exact adjusted *p*-value < 0.05) and then analyzed the set of 152 genes that contained HST bias (Table S3, Additional file 1). Nine of these genes were known imprinting genes, suggesting one parental haplotype could be preferentially silenced [47]. GO analysis of HST-containing genes reveals an enrichment in DNA repair and damage terms (Table S4), an attribute of embryonic stem cells for maintaining genomic integrity [48, 49]. One example from these gene sets is *Mcm5*, a component involved in the DNA helicase complex [50]. The non-reference haplotype that longshot reports corresponds to the *castaneus* parent haplotype [51] and exhibits HST bias (Fig. 1b, c). The *castaneus* haplotype, which contains a variant close to the 5’ splice site of the first exon, is coupled with either splicing with the proximal 5’ splice site or a retained intron; expression from the other haplotype is biased toward isoforms with the distal 5’ splice site. With these results in hybrid mice, we are able to demonstrate FLAIR2’s capacity for incorporating variants in diploid transcriptomes and detecting HSTs in long reads.

### Global downregulation of A-to-I editing following ADAR1 knockdown in short and long reads

**Fig. 2:**
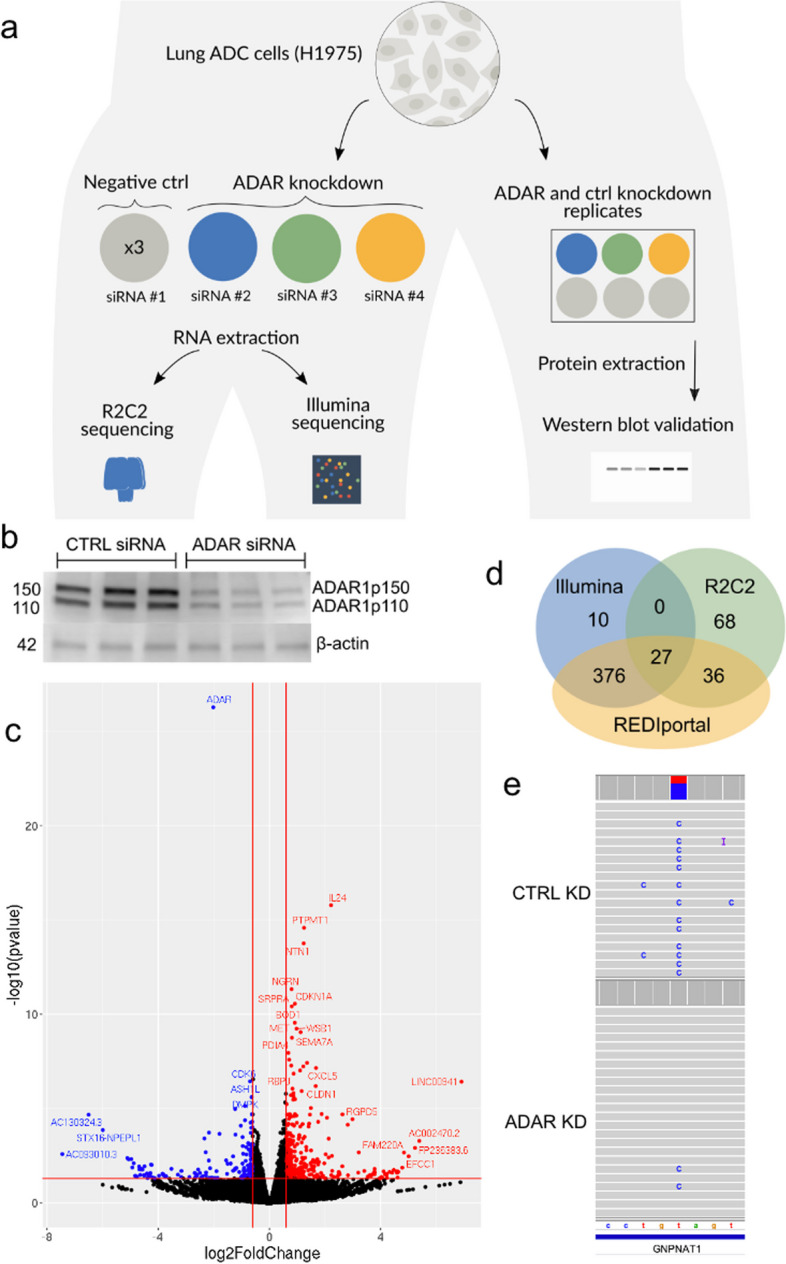
Identification of downregulated inosines with short- and long-read RNA-Seq. **a** Experimental workflow of ADAR knockdown in H1975 cells. **b** Western blot validation of ADAR knockdown. **c** Volcano plot of differentially expressed genes identified from Illumina sequencing. Red: genes with increased expression after ADAR knockdown; blue: genes whose expression went down; black: no change in expression. **d** Venn diagram comparison of the inosines with significant differences in expression identified with Illumina, R2C2 nanopore, or present in the REDIportal database (hg38 liftover). **e** IGV browser view of a downregulated inosine at chr14:52775760 in *GNPNAT1* in the R2C2 data

We applied FLAIR2 to study isoform alterations in relation to inosine editing to build on our understanding of A-to-I editing in the cancer transcriptome. Previous work [10] discovered a connection between A-to-I editing, FAK (PTK2) transcript stability, and increased malignancy using short-read sequencing. We followed their approach of knocking down ADAR and investigating alterations in editing; however, we leveraged the combination of long- and short-read cDNA sequencing (Fig. 2a) to resolve the full-length transcripts edited in lung ADC. First, ADAR1 knockdown was performed in H1975 cells using three different ADAR1 siRNAs (see “Methods”) to achieve 70–80% knockdown of ADAR1 protein levels compared to control replicates (Fig. 2b). We also used a control siRNA to perform a knockdown, generating three technical replicates. Next, we made Illumina short-read RNA-seq sequencing libraries and R2C2 cDNA libraries followed by nanopore sequencing from the same RNA extractions [44]. We observed a 55.1, 73.7, and 78.8% decrease in *ADAR* expression from our normalized Illumina RNA-seq replicates, with *ADAR* being the most significantly downregulated gene (Table S5, Fig. 2c), further confirming *ADAR* knockdown. To reduce nanopore flow cell variability batch effects, each ADAR KD sample was pooled with a control KD sample for a total of three pools of six barcoded samples. Each pool was sequenced on a separate flow cell (Table 1). We obtained an average of 11.7 gigabases (Table 1) with a median raw read length of 9599 bp from each MinION. From the raw basecalled reads, we ran C3POa to call consensus reads (see “Methods”), resulting in error-corrected reads with higher accuracy. We report a median accuracy of 99.3% and a median read length of 1287 bp from our consensus-called reads. As the number of consensus-called and demultiplexed reads provided less power in separate replicates, we decided to combine all of the replicates in each condition together for further analyses.

**Table 1:** R2C2 nanopore sequencing numbers

	Pool 1	Pool 2	Pool 3
Total GB basecalled reads	18.5	7.10	9.56
Number basecalled reads	1,423,603	713,990	778,145
Total GB consensus reads	0.999	0.600	1.03
Median length of consensus reads	1046	1192	1816
Median accuracy (%)	99.2	98.7	99.0
Number aligned CTRL KD consensus reads	445,285	267,746	252,193
Number aligned ADAR KD consensus reads	379,472	184,312	169,506
Number aligned CTRL KD size-selected reads	-	-	6716
Number aligned ADAR KD size-selected reads	-	-	141,754

For each ADAR KD and control KD sample pool that was sequenced on a MinION, we report the total number of reads obtained from sequencing after basecalling, consensus calling, and minimap2 alignment to the hg38 genome. We also show the number of gigabases of reads after basecalling and consensus calling, as well as the median length of the consensus reads. We calculated an accuracy for each read and report the median for each sequencing run

### Inosine detection in short and long reads

We used REDItools [52] to catalog nucleotides at every position in the Illumina data and filtered for the positions that conformed to A-to-I expectations (i.e., positions with an A or T in the reference and read support for G or C). We identified 413 A → G mismatches in the Illumina data that were significantly changed upon ADAR knockdown (Methods), with the majority (403) of these positions present in the REDIportal database [52]. Of the A-to-I events identified with short reads, 409 were downregulated in the knockdown conditions and 4 were upregulated.

**Fig. 3:**
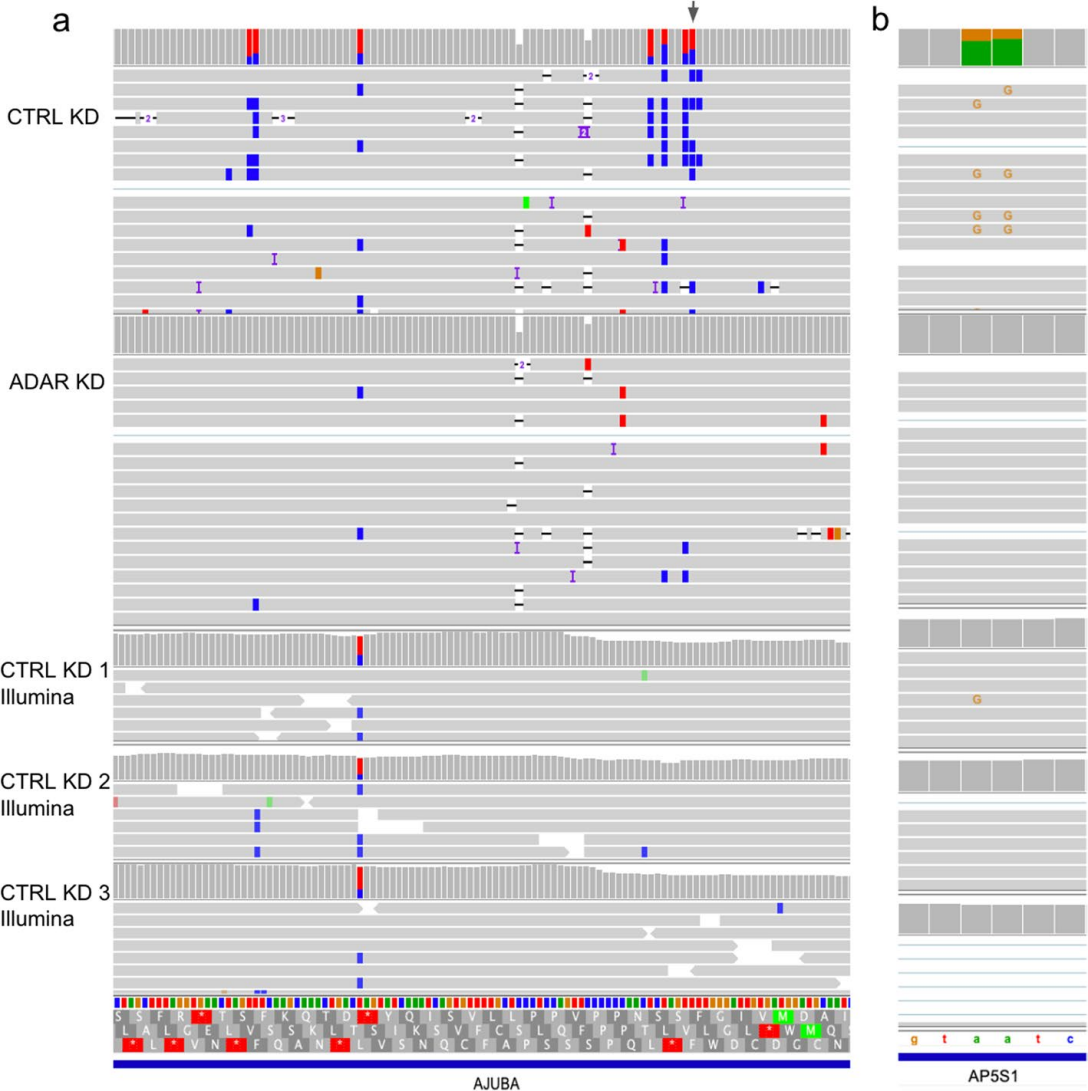
Significantly downregulated A-to-I detected with nanopore and not in the Illumina data. IGV shots of nanopore and Illumina data aligned to hg38. **a** Gray arrow indicates the differentially edited position found in nanopore but not Illumina and is a known editing position in REDIportal. **b** Known A-to-I editing detected by nanopore reads in *AP5S1* but missed in Illumina

We considered longshot and PEPPER-Margin-DeepVariant variant calls to identify an initial set of A-to-G mismatches that we would then reclassify as A-to-I edits with REDIportal and downregulation analyses. Both variant callers identified variants that could be categorized as inosine changes that the other caller missed. Of the variants that overlapped with REDIportal, longshot identified 1230 variants and PEPPER-Margin-DeepVariant identified 3502. We combined all the variant calls from both tools for increased sensitivity, the union resulting in 4020 putative A-to-I events. Starting with the combined variant calls, we identified 63 significantly changed A-to-I events that were also present in REDIportal (Fisher’s exact *p* < 0.05) with a greater than 10% difference in proportion of edited reads (Fig. 2d, Additional file 2). As expected, most (62/63) were downregulated in the ADAR knockdown samples. Of the 131 significant nanopore-identified inosines, 27 were also identified as significantly downregulated in the Illumina data (Fig. 2d, e, Additional file 3). We identified individual bases with a high proportion of editing, defined as type I hyperediting following nomenclature from a previous study which considered bases with > 40% of adenosine residues being edited as type I hyperediting [53]. We found that approximately half (79/131) of the significantly differentially edited inosines were considered type I hyperedited in the control knockdown data. We also noted that 60 of the 68 putative A-to-I events that were identified as significantly differentially edited in only the R2C2 data were downregulated in the knockdown condition. We observed evidence that the lack of discovery of these sites in the Illumina data may be from a lack of coverage. For example, some of the nanopore-only events in the Illumina replicates received insufficient numbers of aligned reads or did not have enough edited reads to pass a significance threshold (Fig. 3, Fig. S3). We computed the coverage of inosines that were called in Illumina data and the coverage of those that were missed by Illumina but found to be significantly knocked down in the R2C2 data and validated with REDIportal, and the latter positions had significantly lower coverage (mean coverage difference of 31.3, Mann–Whitney *U p*-val 0.0176). Of the 68 significant putative inosines found only in nanopore, 53 had reads in at least one control knockdown Illumina sample supporting the edit. To help improve alignments, we aligned unmapped Illumina reads to an edited version of the hg38 reference with specific nucleotides replaced with the nanopore-identified differentially edited sites and found that we were able to increase coverage for 28 sites. In conclusion, while the coverage of short-read data will typically surpass that of long reads lending to an increase in the number of inosines detected, long reads could be advantageous for detecting certain A-to-I events.

### Long reads can identify type II hyperediting

**Fig. 4:**
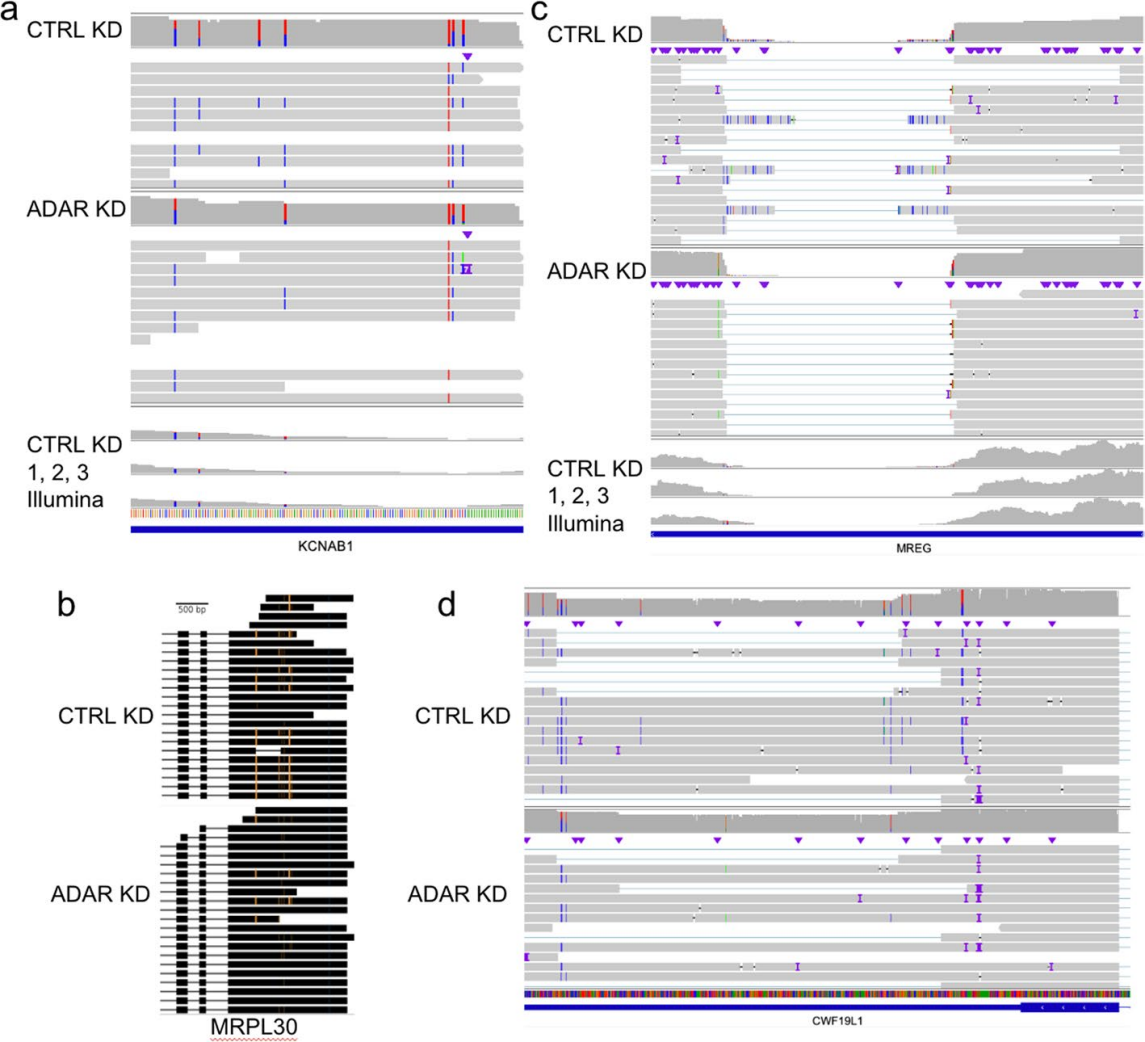
Long-range features of inosines observed with nanopore sequencing. Aligned reads displaying **a** type II hyperediting, **b** coordinated editing, and **c** and **d** disruption of splicing in the presence of editing. In **a** and **c,** the top coverage tracks and reads are displaying the nanopore CTRL/ADAR KD samples, and the bottom three coverage tracks are Illumina CTRL KD samples. In **b** and **d**, the dataset on top displays the control nanopore reads and the bottom panel displays the ADAR knockdown reads. In **b**, orange marks correspond to A → G mismatches and in **a**,** c**, and **d**, positions marked with blue mismatches are T → C mismatches (A → G on the negative strand)

ADAR tends to produce clusters of inosines on a transcript, which we define as type II hyperediting [53]. Hyperedited regions were identified as any window that contained at least three A-to-I edits distributed within every 150 bp—a modified definition derived from Porath et al. [54] which requires > 5% of a short-read’s length to contain A-to-G mismatches. Type II hyperedited transcripts have been associated with nuclear retention or degradation [55–59]. First, we note a pattern of ADAR editing in which transcripts that are edited tend to have multiple edits. The control knockdown data in aggregate show that 38.7% of reads contain at least one edit, and of the reads that are edited, 77.9% contain more than one edit. On detecting multiple edits in short-read RNA-Seq, if the edits are too distant, or if a read contains many mismatches on account of A-to-I hyperediting (type II), reads with multiple edits may not align to the genome and evade detection [54] (Fig. 3). To expand our search space, we used the larger set of all inosines found in our nanopore data and REDIportal that were not necessarily significantly downregulated after knockdown as well as the significantly downregulated inosines discovered with nanopore only. With this approach, we identified 99 regions that overlapped with known type II hyperediting [54] as well as 17 novel hyperedited regions (Fig. 4a).

### Long reads clarify the transcriptional context of inosines

Previous studies have established a connection between editing and changes in splicing, either in *cis* or *trans* [14]. However, we were not able to find many convincing cases of alternative splicing from ADAR knockdown alone with the Illumina data. We ran the differential splicing analysis tools juncBASE [60] and JUM [61] (see “Methods”). None of the identified splicing events was significant after multiple testing corrections. With our nanopore data, we sought to find edits associated with the presence of other edits or splicing changes that could be overlooked in the Illumina data due to potential mapping difficulties or length limitations. We performed a systematic analysis of all inosine-inosine associations within single molecule reads [62]. For each inosine, we looked at the nearest 20 variants, checked all of the reads that overlapped both variants to count the frequency they co-occured with each other, and performed a Fisher’s test to discover significantly associated positions. We observed 12 associated inosines that satisfied these conditions with a Fisher’s exact *p*-value < 0.05. In *MRPL30*, we noted coordinated inosine editing occurring more than 500 bp apart within Alu elements (*p* = 2.35e − 6) (Fig. 4b). The predicted secondary structure of the 3′ UTR consists of a hairpin that can form between them, potentially bringing the two sites in closer proximity (Fig. S4). We also noticed a pattern in the 3′ UTR of melanoregulin (*MREG*) transcripts whereby splicing alterations appeared to be coordinated with A-to-I edits. Our nanopore data show that for splicing within the 3′ UTR of *MREG*, there are several positions proximal to splice sites that are edited and unspliced in the CTRL KD samples (Fig. 4c). The STAR short-read aligner did not report these splice junctions and there is a lack of Illumina reads aligning across this *MREG* splice site. We then looked for other genes that demonstrated the same mutually exclusive pattern of reads either containing an inosine or having an intron spliced out. We found 145 hyperedited sites that resided within introns of other reads assigned to that gene (Additional file 4). Three of these sites can be found in the 3′ UTR of *CWF19L1* (Fig. 4d). These cases illustrate the ways in which long reads can provide benefits over short reads, drawing connections between A-to-I edits where length limitations would affect short-read analyses.

## Discussion

The additive complexity of RNA editing and splicing on the transcriptome, in addition to the disease implications of aberrations in these processes, necessitate methods for more thorough profiling of RNA transcripts. We sought to bridge our understanding of A-to-I editing using short- and long-read sequencing to identify edits more extensively as well as investigate any events that require the full transcriptional context to decipher. We knocked down ADAR in lung adenocarcinoma cells and sequenced the cDNA with the more accurate R2C2 nanopore sequencing method. We were able to discover putative novel type I and type II hyperediting (Figs. 2e and 3a), sites that are coordinated with each other (Fig. 4b), and sites that may disrupt splicing (Fig. 4c, d). We observed a general decrease in Illumina coverage of the novel nanopore sites that are missed in short-read data, indicating potential alignment challenges for short reads with many A-to-I mismatches such that only unedited reads align. While such sites may not have been identified by Illumina due to coverage issues, many sites could still be corroborated with short-read realignments, increasing our confidence in them. While we considered novel, putative inosines detected only in R2C2 data as those that we observed significant downregulation of upon ADAR knowndown, additional validation is necessary to distinguish true novel inosines from false positives arising from any homopolymer or systematic errors. 

Previous work in Alzheimer’s disease has found similar patterns of coupled enriched A-to-I editing in isoforms with longer 3′ UTRs [63]. In this study, we found cases where 3′ UTRs were spliced or edited in a mutually exclusive manner. From another study, ADAR-dependent editing of the 3′ UTR has been observed to increase expression [64]. This suggests that the elevated levels of editing present in H1975 cells could promote expression of those transcripts bearing edits in their 3′ UTRs. The documentation of these transcripts that are targets of ADAR and alternative splicing brings attention to further efforts to disentangle these regulatory processes involved in the cancer transcriptome.

We have observed that HST detection is feasible with R2C2 long reads in the LRGASP mouse and the H1975 ADAR knockdown data. The performance of HST detection with FLAIR2 on data with lower quality than R2C2, such as with direct RNA sequencing, may be challenging and must be further explored. It should be noted that FLAIR2 itself is not a variant caller, but can integrate variants called from other tools or vetted by other sources to assemble into HSTs. The quality of variant calls impacts FLAIR2’s ability to determine HSTs, and error-prone data may not be recommended for variant calling due to higher chances of false positive calls. As the quality of long reads and the tools for variant calling in long reads, including for A-to-I edits, continue to improve [65–68], the limitations of HST detection in long-read data may decrease.

Future studies would benefit from the selection of longer molecules to sequence incompletely spliced RNAs and thus capture more unspliced, intron-containing transcripts to reveal more into the regulation of intronic A-to-I editing. Nevertheless, we were still able to build a computational tool to leverage our accurate nanopore data in ways that surpassed the limitations of short reads, continuing to pave a way for the adoption of long reads for characterizing RNA splicing and editing in cancers.

## Methods

### Cell culture and siRNA knockdown

H1975 cells were cultured in T75 flasks with DMEM + 10% FBS media. Cells were split 1:4 every 3 days using a 0.25% trypsin 0.52 mM EDTA solution. Trypsin solution was neutralized using an approximately equal volume of media.

For ADAR and control knockdowns, we used one of the Thermo Fisher Silencer Select siRNAs s1007, s1008, and s1009 for the three *ADAR1* biological replicates and Silencer Select Negative Control No. 1 at 15 nmol for 72 h. Cells that would be subject to RNA extraction were cultured in 10-cm dishes. In tandem, cells were plated for western blotting in 6-well plates. Depending on the vessel media volume, the appropriate amount of siRNA was added to the media when the cells were 80% confluent.

### Western blotting

We have uploaded our Western blotting protocol to protocols.io [69]. Briefly, after siRNA treatment, the media were aspirated off and 200 ul of cold RIPA and proteinase K solution were added to each well. Cells were scraped off, transferred to cold tubes, and centrifuged at 15,000 × g for 7 min. Leaving the pellet, the supernatant lysate was then retained in protein lo-bind tubes. Protein lysates were sonicated twice for 30 s, with 1 min on ice in between rounds of sonication. Protein concentrations were measured with the Pierce BCA Protein Assay. According to the concentrations to ensure approximately equivalent amounts of protein, the lysates were loaded into Bio-rad Mini-PROTEAN precast gels. We used ADAR1 primary antibody (Abcam ab88574) and goat secondary (Abcam ab205719) and imaged on LI-COR C-digit blot scanner. Relative protein abundance levels were measured from blot images using FIJI software to calculate degree of knockdown with ACTB as the loading controls to normalize.

### RNA extraction

Media was aspirated off and the dishes were washed 3 × with ice-cold dPBS. 1 ml of tri-reagent was added to each dish and cells were scraped off. Cells suspended in tri-reagent were used as input into the Zymo Direct-zol kit. Following elution from the Direct-zol kit, RNA quality, and concentration were evaluated with a Nanodrop, Qubit, and Tapestation (RIN 9.5–9.8).

### R2C2 cDNA size selection

The first ADAR KD and CTRL KD replicates’ R2C2 libraries were size selected for longer fragments; longer fragments will contain more repeats of the original insert sequence, thereby increasing the accuracy of the consensus reads. To size select, bead purified cDNA was combined and then run on a 1% low-melt agarose gel made with TAE (tris–acetate-EDTA). A gel slice containing cDNA above 3 kb was cut out and placed in twice the volume of beta-agarase buffer, incubating on ice. The buffer was refreshed after 20 min. After another 20 min, the buffer was removed and the gel was melted at 65 °C for 10 min. The gel was then incubated overnight with the addition of 2 µl of beta-agarase per 300 µl of gel. A bead purification was performed on the DNA-containing digested gel.

### R2C2 library preparation, paired sample pooling, and nanopore sequencing

We followed the R2C2 protocol from Vollmers et al. [70], with a protocol also shared online (https://docs.google.com/document/d/1IyZnNTd2KBElJ2qZdDgE2TVue3aEggeDT8nfHt8qDKc/edit). We also have the workflow written out on protocols.io as adapted for this work (dx.doi.org/10.17504/protocols.io.n2bvjx3knlk5/v1). In summary, our steps were as follows: Each ADAR KD sample was pooled with a control KD sample for a total of three pools for six samples, each pool to be sequenced on a separate flow cell. On the first flow cell, the ADAR KD replicate #1 and CTRL KD replicate #1 were pooled and sequenced together as Pool 1, and so on for the other two replicates for each knockdown condition and flow cells. R2C2 library preparation for the pools started from RNA extracted from H1975 cells. First, RNAs from each sample were reverse transcribed with SmartSeq and barcoded oligo-dTs. For Pools 1 and 2, 1 µg of RNA was used for the RT step, and for Pool 3, 200 ng was used. The oligo-dT index sequences used for the R2C2 protocol are included in Table S6 and the full splint sequences are on the Vollmers’ lab R2C2 protocol document. The RT product underwent lambda exonuclease and RNase A digestion, followed by 15 cycles of PCR using KAPA Hifi HotStart ReadyMix. Next, the cDNA was cleaned with 0.8:1 ampure bead purification. The Pool 2 samples were cleaned using Zymo Select-A-Size for fragments larger than 300 nt, adding an extra empty spin step after the second wash. Samples were then circularized and amplified to form long R2C2 concatemers. For Pool 3, before subjecting the R2C2 libraries to nanopore sequencing preparation, a size selection step was performed. Pool 3 consisted of size-selected s1007 ADAR KD and one of the CTRL KD replicates combined with the same two samples without size selection. The size selection was to retain concatemeric fragments larger than 3 kb using a low-melt agarose gel extraction. R2C2 cDNA concentration was assessed on a Nanodrop and Qubit prior to nanopore sequencing preparation per ONT 1D sequencing protocol.

Completed nanopore libraries were quantified with Qubit and only 200 ng of the nanopore library were initially loaded onto each flow cell to start sequencing. Excess library was stored at 4 °C. After 24 h, any remaining library was loaded after flushing the flow cell with Nuclease flush buffer and DNAse I according to ONT protocol. Reads were basecalled with guppy 4.4.1 and consensus was called and demultiplexed using C3POa v2.2.3. From the basecalled raw reads that contain multiple passes over the original circularized read, C3POa identifies and combines repeats, or subreads [70]. We used these error-corrected consensus reads for further analyses.

### FLAIR2 splice site fidelity checking and novel isoform detection

FLAIR2 was used for this study, starting with alignment to the genome (hg38 for human, mm10 for mouse) and read correction using the *align* and *correct* modules. While new options for users have been added, the *align* and *correct* module core algorithms have not changed. Annotated splice junctions were used for the alignment step, specified with –junction_bed. The short-read junctions were used in the lung H1975 R2C2 FLAIR2 run where we had matched Illumina data, called from STAR alignments of the Illumina data and specified to FLAIR *correct* with -j. FLAIR2’s updates to isoform detection from the previous version are the splice site fidelity checking and annotation-reliant additional alignment that can be specified in the *collapse* module. We used the –annotation_reliant argument in the FLAIR2 *collapse* module to invoke the additional transcript alignment step to GENCODE v38 annotation for improved isoform detection. The FLAIR2 collapse module first performs an ungapped alignment of reads to transcripts. The top transcript alignments for each read, as determined by minimap2 [71] mapping quality score, are examined with increased stringency: we used the –stringent and –check_splice parameters in FLAIR *collapse* to improve the accuracy of read-isoform assignments, particularly around splice sites. The --stringent parameter enforces that 80% of bases match between the read and assigned isoform as well as that the read spans into 25 bp of the first and last exons. The --check_splice parameter enforces that 4 out of 6 bases flanking every splice site in the transcript are matched in a given read and that there are no indels larger than 3 bp at a splice site. In full, the command we used to run FLAIR2 *collapse* for the SIRVs and ADAR KD R2C2 data is *python flair.py collapse --check_splice --stringent -s 3 -f ref_annotation.gtf -g ref_genome.fasta --generate_map -q flair_corrected.bed --annotation_reliant generate -r reads.fastq*. These new options are incorporated in FLAIR v.1.5.1 and above; subsequent improvements have been made to test the code and make it more user-friendly.

Reads that match annotated isoforms in accordance with these parameters are attached to the isoform as a supporting read. These annotated isoforms with read support are included in the final set of FLAIR isoforms. The remaining, unassigned reads are used for novel isoform detection. The process of summarizing the unassigned reads into the isoforms begins with minimap2 to align the reads to the genome. FLAIR corrects unsupported splice sites with the closest splice site that contains more evidence i.e., splice sites found in annotations or short-read sequencing. The corrected reads are then grouped by their splice junction chains. For each group, FLAIR calls transcription start and end sites, collapsing each group into one or more representative first-pass isoform. These default parameters for calling end sites are a maximum of two TSSs and TESs picked per splice junction chain, with these positions picked based on those that are most frequent using a fuzzy window. Next, FLAIR assigns each read to a first-pass isoform by realigning the reads to the isoforms and identifying the best alignment with the splice site fidelity stringency previously specified. The final FLAIR isoform set arises from filtering the first-pass set for the novel isoforms that pass a minimum supporting read threshold combined with the annotated isoforms.

### SIRV analysis

The SIRV set used was Lexogen SIRV Set 4 as documented for the LRGASP study (https://lrgasp.github.io/lrgasp-submissions/docs/reference-genomes.html). These are 69 synthetic RNA transcripts on the SIRV1-7 chromosomes made to mimic a complex human transcriptome with genes that are alternatively spliced. Each transcript has on average 5.17 exons with 316 unique exons shared between these transcripts. We analyzed SIRV reads that aligned with the SIRV1-SIRV7 references from the LRGASP mouse embryonic stem cell R2C2 sequencing replicates [40]. We ran FLAIR2 providing the complete genome annotation and with the default minimum supporting read count of 3 (-s). We used the -L parameter and supplied a genome annotation for the stringtie2 v2.0 run, using the default parameters otherwise. For FLAMES (cloned from GitHub on June 1, 2021), we used the provided SIRV config file and altered the minimum supporting read count of 3. We used gffcompare [72] to calculate transcript-level sensitivity and precision of each tool’s transcript reference with the ground truth, using a wiggle room of 50 bp at the transcription start sites and terminal ends for matching (-e 50 and -d 50).

### LRGASP R2C2 Sequencing Benchmarks and Evaluation

LRGASP WTC-11 R2C2 sequencing (non-size-selected) data were obtained from the ENCODE DCC (https://www.encodeproject.org/) from accession numbers ENCFF089IVT, ENCFF548RZB, ENCFF997UNC. SIRV-set4 sequences were obtained from these files as well. Matched Illumina short-read sequencing was obtained from accession number ENCSR673UKZ. Sequencing from biological replicates were combined. FLAIR2 was run using default parameters except the flair collapse module was run with –annotation_reliant, --check_splice, and --stringent parameters. Reported performance results for Bambu, IsoQuant, Lyric, Mandalorion, and Talon were obtained from the LRGASP Consortium. For evaluation, LRGASP CAGE-seq (GEO GSE185917) and Quant-seq (GEO GSE219685) were also obtained. The version of SQANTI3 used for LRGASP evaluation (https://github.com/LRGASP/lrgasp-challenge-1-evaluation/) was used to evaluate the FLAIR2 WTC11 transcripts against the GENCODE v38 reference or a GENCODE manual annotation provided by LRGASP. The %CAGE and %Quant were calculated from the total supported isoforms (FSM+ISM+NIC+NNC) divided by the total isoforms from the SQUANTI report html file. The %SRTM was calculated as all SRTM transcripts divided by the total FSM+ISM. The %SNTM was calculated as all SNTM transcripts divided by the total NIC+NNC. The %SJ cov was calculated by dividing the number of junctions from the SQANTI_junctions.txt file with >=1 read support in short reads by the total junctions in that file. The gencode sensitivity and precision for known and novel transcripts was based off of the subset of transcripts verified by gencode and was determined by running the code from https://github.com/LRGASP/Challenge1_Figures_Code/ for supplementary figure 34.

### Variant integration into FLAIR isoforms and read simulation

We ran two long-read variant callers on our data. Longshot was run with default and required arguments in addition to --min_allele_qual set to 3 and the -F argument. Pepper-Margin-DeepVariant version r0.7 was run on a bam file with all cigar string N operators changed to D and H operators removed. We ran Pepper-Margin-DeepVariant with default and required arguments including the --ont_r9_guppy5_sup argument. For the longshot-phased version of FLAIR, we supplied FLAIR-collapse the longshot bam and vcf output using the --longshot_bam and --longshot_vcf arguments, respectively. The longshot bam contains aligned reads with an additional phase set and haplotype tag attached to each read, which can then be counted to see which isoforms have a majority fraction of haplotype-assigned reads. For the variant caller-agnostic method, we filtered the vcfs generated from longshot and pepper by coverage and then combined them. This vcf, along with FLAIR-collapse isoform output, was supplied to a FLAIR script called assign_variants_to_isoforms. Both methods of variant integration ultimately yield a sequence fasta file with the variant-containing isoform sequences, an updated isoform model file, as well as a vcf of the variants and the isoform names that contain those variants.

Reads were simulated to test FLAIR2’s multi-haplotype isoform detection with assign_variants_to_isoforms. Complex editing haplotype sequences were manually created. Reads were simulated using badread [73] with the following command: badread-runner.py simulate --reference ref_sequences.fa --quantity 15x --glitches 10000,10,10 --junk_reads 0.1 --random_reads 0.1 --chimeras 0.1 --identity 20,3. The simulated reads as well as the known simulated variant positions were provided to FLAIR2’s assign_variants_to_isoforms submodule to call HSTs with the command. The isoforms and the different haplotypes represented in each isoform are output and the isoform structures with variants can be simultaneously visualized in IGV after aligning the HST sequences.

### Illumina RNA-seq analysis

Illumina reads were aligned to the hg38 genome using hisat2 v2.1.0 [74]. Genes counts were calculated using htseq-count [75] given the aligned bams and then passed to DESeq2 v1.22.2 [76] for differential gene expression analysis using the default parameters. The level of ADAR knockdown in each replicate was calculated by comparing the normalized level of ADAR expression in short reads in each control knockdown replicate with its corresponding ADAR knockdown replicate (same-numbered replicate).

For editing analysis, REDItools [52] was used to tabulate the number of reads supporting each base at every position. The REDItools output was filtered using custom python scripts for positions that contained guanosine mismatches at positions where the reference base was an adenosine for genes corresponding to the forward strand of the genome, and the reverse complement for those on the reverse strand. Positions were considered putatively edited if the SNV was G → A or T → C, and positions with less than 15% of reads representing the edited base were filtered out. The counts of the reference and alternate allele in each of the samples for the remaining positions were supplied to DRIMSeq [77] for differential testing between two conditions, with the settings that at least 5 reads contained editing (G mismatch) in a minimum of two samples, as well as a coverage of 15 reads minimum in at least 3 samples. For splicing identification, we ran juncBASE v1.2-beta following the manual with default parameters. We ran jum v2.0.2 with the parameters `--JuncThreshold 5 --Condition1_fileNum_threshold 2 --Condition2_fileNum_threshold 2` and default parameters otherwise.

### Inosine detection in long reads and inosine coordination analysis

We used the python package pysam’s pileup method to count A → G or T → C reads at all positions in the nanopore data identified from variant calling. Next, we combined counts of either allele from the control knockdown replicates together or the ADAR knockdown replicates together. We performed a Fisher’s exact test using the number of unedited and edited reads in the ADAR knockdown or control knockdown to assess the significance of the A-to-I differences. After applying multiple testing corrections to these *p*-values, few events were significant so we only considered A-to-I discovery in the nanopore data as those with uncorrected *p*-values < 0.05. We filtered for positions that had a minimum coverage of 10 in either condition and a change in percentage of edited reads after ADAR knockdown of 10% or more.

For the long-range inosine coordination analysis to test for inosines that were more frequently edited together, we first considered inosines that were at least 50 bp apart. The number of edited and unedited reads at each position was assessed for significance with a Fisher’s exact test and this was repeated for all pairs of inosines that appeared on the same molecules. We looked at the secondary structure of *MPRL30* by inputting part of its 3′ UTR sequence including the two coordinated inosines to the RNAStructure web server [78]. For the inosine-intron coordination analysis, we filtered for sites that were type I hyperedited (i.e., more than 40% of residues were edited) and had at least 10 reads that were edited and at least 10 reads where that position was spliced out, i.e., fell within an intron for that aligned read.

### Supplementary Information

Additional file 1. HSTs identified in hybrid Castaneus x Mouse 129. Columns are as follows: 1) chromosome; 2) position; 3) reference allele; 4) alternate allele; 5) FLAIR isoform containing ENSMUSG gene id; 6) p-value of Fisher’s test; the number of reads supporting the 7) reference or 8) alternate allele in the transcript in column 5; the number of reads supporting the 9) reference or 10) alternate allele in all other transcripts of the same gene.

Additional file 2. Significant A-to-I changes identified in R2C2 nanopore data. Columns are as follows: 1) chromosome and 2) position of inosines, the number of reads in the control KD sample that are 3) not edited or 4) edited, the number of reads in the ADAR KD samples that are 5) not edited or 6) edited, and the 7) Fisher’s exact test *p*-value.

Additional file 3. Cross-platform comparison of A-to-I edits identified. Columns are as follows: 1) chromosome and 2) position of inosine, whether or not the position was detected in 3) Illumina; 4) Illumina but not significantly downregulated upon ADAR knockdown; 5) nanopore; 6) nanopore and was significantly downregulated upon ADAR knockdown; 7) present in REDIportal.

Additional file 4. A-to-I editing within intronic regions. Columns are as follows: 1) chromosome and 2) position of inosines, the number of 3) unedited reads, 4) edited reads, and 5) number of reads with the inosine spliced out.

Additional file 5. Figures S1-S4.

Additional file 6: Table S1. Performance of transcript detection tools on 1D nanopore SIRVs. Table S2. Performance of transcript detection tools on R2C2 nanopore SIRV-Set4 from the LRGASP Consortium. Performance of Bambu, IsoQuant, Lyric, Mandalorion, and TALON were obtained from Pardo-Palacios et al. Table S3. HST-containing genes in hybrid Castaneus x Mouse 129 identified from this study. Table S4. GO terms associated with HSTs found in hybrid Castaneus x 129 mouse data. Table S5. DESeq2 output table of differentially expressed genes upon KD of ADAR in H1975. Analysis is from Illumina sequencing data. Table S6. Oligo-dT indexes used with R2C2 sequencing.

Additional file 7. Peer review history.

## Data Availability

The R2C2 consensus read sequences have been submitted to SRA under bioproject PRJNA981664 [79]. FLAIR2 isoforms with longshot variants have been uploaded to Zenodo at https://zenodo.org/record/8019704 [80] and https://zenodo.org/records/11399236 [81]. FLAIRv2.0 is available on GitHub at https://github.com/BrooksLabUCSC/flair and detailed documentation can be found at https://flair.readthedocs.io [82]. The FLAIR v2.0 release can also be found at https://zenodo.org/records/11289665 [83].

## References

[1] Athanasiadis A, Rich A, Maas S (2004). Widespread A-to-I RNA editing of Alu-containing mRNAs in the human transcriptome. PLoS Biol.

[2] Levanon EY, Eisenberg E, Yelin R, Nemzer S, Hallegger M, Shemesh R (2004). Systematic identification of abundant A-to-I editing sites in the human transcriptome. Nat Biotechnol.

[3] Nishikura K (2010). Functions and regulation of RNA editing by ADAR deaminases. Annu Rev Biochem.

[4] Kiran AM, O’Mahony JJ, Sanjeev K, Baranov PV (2013). Darned in 2013: inclusion of model organisms and linking with Wikipedia. Nucleic Acids Res.

[5] Bazak L, Haviv A, Barak M, Jacob-Hirsch J, Deng P, Zhang R (2014). A-to-I RNA editing occurs at over a hundred million genomic sites, located in a majority of human genes. Genome Res.

[6] Sommer B, Köhler M, Sprengel R, Seeburg PH (1991). RNA editing in brain controls a determinant of ion flow in glutamate-gated channels. Cell.

[7] Burnashev N, Monyer H, Seeburg PH, Sakmann B (1992). Divalent ion permeability of AMPA receptor channels is dominated by the edited form of a single subunit. Neuron.

[8] Bajad P, Jantsch MF, Keegan L, O’Connell M (2017). A to I editing in disease is not fake news. RNA Biol.

[9] Han L, Diao L, Yu S, Xu X, Li J, Zhang R (2015). The Genomic Landscape and Clinical Relevance of A-to-I RNA Editing in Human Cancers. Cancer Cell.

[10] Amin EM, Liu Y, Deng S, Tan KS, Chudgar N, Mayo MW, et al. The RNA-editing enzyme ADAR promotes lung adenocarcinoma migration and invasion by stabilizing FAK. Sci Signal. 2017;10. 10.1126/scisignal.aah3941. 10.1126/scisignal.aah3941 PMC5771642 28928239

[11] Lazzari E, Mondala PK, Santos ND, Miller AC, Pineda G, Jiang Q (2017). Alu-dependent RNA editing of GLI1 promotes malignant regeneration in multiple myeloma. Nat Commun.

[12] Rueter SM, Dawson TR, Emeson RB (1999). Regulation of alternative splicing by RNA editing. Nature.

[13] Hsiao Y-HE, Bahn JH, Yang Y, Lin X, Tran S, Yang E-W (2018). RNA editing in nascent RNA affects pre-mRNA splicing. Genome Res.

[14] Tang SJ, Shen H, An O, Hong H, Li J, Song Y (2020). Cis- and trans-regulations of pre-mRNA splicing by RNA editing enzymes influence cancer development. Nat Commun.

[15] Pan Q, Shai O, Lee LJ, Frey BJ, Blencowe BJ (2008). Deep surveying of alternative splicing complexity in the human transcriptome by high-throughput sequencing. Nat Genet.

[16] Chen CX, Cho DS, Wang Q, Lai F, Carter KC, Nishikura K (2000). A third member of the RNA-specific adenosine deaminase gene family, ADAR3, contains both single- and double-stranded RNA binding domains. RNA.

[17] Mannion NM, Greenwood SM, Young R, Cox S, Brindle J, Read D (2014). The RNA-editing enzyme ADAR1 controls innate immune responses to RNA. Cell Rep.

[18] Liddicoat BJ, Piskol R, Chalk AM, Ramaswami G, Higuchi M, Hartner JC (2015). RNA editing by ADAR1 prevents MDA5 sensing of endogenous dsRNA as nonself. Science.

[19] Pestal K, Funk CC, Snyder JM, Price ND, Treuting PM, Stetson DB (2015). Isoforms of RNA-editing enzyme ADAR1 independently control nucleic acid sensor MDA5-driven autoimmunity and multi-organ development. Immunity.

[20] Li Q, Gloudemans MJ, Geisinger JM, Fan B, Aguet F, Sun T (2022). RNA editing underlies genetic risk of common inflammatory diseases. Nature.

[21] Kawahara Y, Kwak S, Sun H, Ito K, Hashida H, Aizawa H (2003). Human spinal motoneurons express low relative abundance of GluR2 mRNA: an implication for excitotoxicity in ALS: AMPA subunit expression profile in human CNS. J Neurochem.

[22] Livingston JH, Lin J-P, Dale RC, Gill D, Brogan P, Munnich A (2014). A type I interferon signature identifies bilateral striatal necrosis due to mutations in ADAR1. J Med Genet.

[23] Rice GI, Kasher PR, Forte GMA, Mannion NM, Greenwood SM, Szynkiewicz M (2012). Mutations in ADAR1 cause Aicardi-Goutières syndrome associated with a type I interferon signature. Nat Genet.

[24] Gumireddy K, Li A, Kossenkov AV, Sakurai M, Yan J, Li Y (2016). The mRNA-edited form of GABRA3 suppresses GABRA3-mediated Akt activation and breast cancer metastasis. Nat Commun.

[25] Chen L, Li Y, Lin CH, Chan THM, Chow RKK, Song Y (2013). Recoding RNA editing of AZIN1 predisposes to hepatocellular carcinoma. Nat Med.

[26] Ramaswami G, Li JB (2014). RADAR: a rigorously annotated database of A-to-I RNA editing. Nucleic Acids Res.

[27] Picardi E, Manzari C, Mastropasqua F, Aiello I, D’Erchia AM, Pesole G (2015). Profiling RNA editing in human tissues: towards the inosinome Atlas. Sci Rep.

[28] Volden R, Palmer T, Byrne A, Cole C, Schmitz RJ, Green RE (2018). Improving nanopore read accuracy with the R2C2 method enables the sequencing of highly multiplexed full-length single-cell cDNA. Proc Natl Acad Sci U S A.

[29] Byrne A, Supple MA, Volden R, Laidre KL, Shapiro B, Vollmers C (2019). Depletion of hemoglobin transcripts and long-read sequencing improves the transcriptome annotation of the polar bear (Ursus maritimus). Front Genet.

[30] Tang AD, Soulette CM, van Baren MJ, Hart K, Hrabeta-Robinson E, Wu CJ (2020). Full-length transcript characterization of SF3B1 mutation in chronic lymphocytic leukemia reveals downregulation of retained introns. Nat Commun.

[31] Kovaka S, Zimin AV, Pertea GM, Razaghi R, Salzberg SL, Pertea M (2019). Transcriptome assembly from long-read RNA-seq alignments with StringTie2. Genome Biol.

[32] Tian L, Jabbari JS, Thijssen R, Gouil Q, Amarasinghe SL, Voogd O (2021). Comprehensive characterization of single-cell full-length isoforms in human and mouse with long-read sequencing. Genome Biol.

[33] Wyman D, Balderrama-Gutierrez G, Reese F, Jiang S, Rahmanian S, Zeng W, et al. A technology-agnostic long-read analysis pipeline for transcriptome discovery and quantification. bioRxiv. 2019. p. 672931. Available from: https://www.biorxiv.org/content/10.1101/672931v1. [cited 2020 Feb 7].

[34] Volden R, Schimke KD, Byrne A, Dubocanin D, Adams M, Vollmers C (2023). Identifying and quantifying isoforms from accurate full-length transcriptome sequencing reads with Mandalorion. Genome Biol.

[35] van Baren J, Brooks AN, Tang A. FLAIR2. Code respository. 2023. Available from: https://zenodo.org/records/11289665.

[36] Glinos DA, Garborcauskas G, Hoffman P, Ehsan N, Jiang L, Gokden A (2022). Transcriptome variation in human tissues revealed by long-read sequencing. Nature.

[37] Deonovic B, Wang Y, Weirather J, Wang X-J, Au KF (2017). IDP-ASE: haplotyping and quantifying allele-specific expression at the gene and gene isoform level by hybrid sequencing. Nucleic Acids Res.

[38] Workman RE, Tang AD, Tang PS, Jain M, Tyson JR, Razaghi R (2019). Nanopore native RNA sequencing of a human poly(A) transcriptome. Nat Methods.

[39] Liu B, Liu Y, Li J, Guo H, Zang T, Wang Y (2019). deSALT: fast and accurate long transcriptomic read alignment with de Bruijn graph-based index. Genome Biol.

[40] Pardo-Palacios FJ, Wang D, Reese F, Diekhans M, Carbonell-Sala S, Williams B, et al. Systematic assessment of long-read RNA-seq methods for transcript identification and quantification. Nat Methods. 2024. 10.1038/s41592-024-02298-3. 10.1038/s41592-024-02298-3 PMC11543605 38849569

[41] Chen Y, Sim A, Wan YK, Yeo K, Lee JJX, Ling MH (2023). Context-aware transcript quantification from long-read RNA-seq data with Bambu. Nat Methods.

[42] Prjibelski AD, Mikheenko A, Joglekar A, Smetanin A, Jarroux J, Lapidus AL (2023). Accurate isoform discovery with IsoQuant using long reads. Nat Biotechnol.

[43] Carbonell-Sala S, Lagarde J, Nishiyori H, Palumbo E, Arnan C, Takahashi H, et al. CapTrap-Seq: A platform-agnostic and quantitative approach for high-fidelity full-length RNA transcript sequencing. bioRxiv. 2023. 10.1101/2023.06.16.543444. 10.1038/s41467-024-49523-3 PMC11211341 38937428

[44] Tang AD, Felton C, Brooks AN (2024). FLAIR2 results from LRGASP R2C2 sequencing of Human WTC11 and Mouse ES cell lines. Datasets. Repository. Detecting haplotype-specific transcript variation in long reads with FLAIR2.

[45] Edge P, Bansal V (2019). Longshot enables accurate variant calling in diploid genomes from single-molecule long read sequencing. Nat Commun.

[46] Shafin K, Pesout T, Chang P-C, Nattestad M, Kolesnikov A, Goel S (2021). Haplotype-aware variant calling with PEPPER-Margin-DeepVariant enables high accuracy in nanopore long-reads. Nat Methods.

[47] Santini L, Halbritter F, Titz-Teixeira F, Suzuki T, Asami M, Ma X (2021). Genomic imprinting in mouse blastocysts is predominantly associated with H3K27me3. Nat Commun.

[48] Fu X, Cui K, Yi Q, Yu L, Xu Y (2017). DNA repair mechanisms in embryonic stem cells. Cell Mol Life Sci.

[49] Sun S, Osterman MD, Li M (2019). Tissue specificity of DNA damage response and tumorigenesis. Cancer Biol Med.

[50] Forsburg SL (2004). Eukaryotic MCM proteins: beyond replication initiation. Microbiol Mol Biol Rev.

[51] Harr B, Karakoc E, Neme R, Teschke M, Pfeifle C, Pezer Ž (2016). Genomic resources for wild populations of the house mouse, Mus musculus and its close relative Mus spretus. Sci Data.

[52] Mansi L, Tangaro MA, Lo Giudice C, Flati T, Kopel E, Schaffer AA (2021). REDIportal: millions of novel A-to-I RNA editing events from thousands of RNAseq experiments. Nucleic Acids Res.

[53] Tavakoli S, Nabizadeh M, Makhamreh A, Gamper H, McCormick CA, Rezapour NK (2023). Semi-quantitative detection of pseudouridine modifications and type I/II hypermodifications in human mRNAs using direct long-read sequencing. Nat Commun.

[54] Porath HT, Carmi S, Levanon EY (2014). A genome-wide map of hyper-edited RNA reveals numerous new sites. Nat Commun.

[55] Prasanth KV, Prasanth SG, Xuan Z, Hearn S, Freier SM, Bennett CF (2005). Regulating gene expression through RNA nuclear retention. Cell.

[56] Scadden ADJ (2005). The RISC subunit Tudor-SN binds to hyper-edited double-stranded RNA and promotes its cleavage. Nat Struct Mol Biol.

[57] Scadden ADJ (2007). Inosine-containing dsRNA binds a stress-granule-like complex and downregulates gene expression in trans. Mol Cell.

[58] Hundley HA, Krauchuk AA, Bass BLC (2008). elegans and H. sapiens mRNAs with edited 3’ UTRs are present on polysomes. RNA.

[59] Chen L-L, Carmichael GG (2009). Altered nuclear retention of mRNAs containing inverted repeats in human embryonic stem cells: functional role of a nuclear noncoding RNA. Mol Cell.

[60] Brooks AN, Yang L, Duff MO, Hansen KD, Park JW, Dudoit S (2011). Conservation of an RNA regulatory map between Drosophila and mammals. Genome Res.

[61] Wang Q, Rio DC (2018). JUM is a computational method for comprehensive annotation-free analysis of alternative pre-mRNA splicing patterns. Proc Natl Acad Sci U S A.

[62] Tang A, Brooks AN. Long read isoforms with A-to-I edits for H1975 cell line. Datasets. Repository. Detecting haplotype-specific transcript variation in long reads with FLAIR2. 2023. Available from: https://zenodo.org/records/8019704. 10.1186/s13059-024-03301-y PMC11218413 38956576

[63] Course MM, Gudsnuk K, Keene CD, Bird TD, Jayadev S, Valdmanis PN. Aberrant splicing of PSEN2, but not PSEN1, in individuals with sporadic Alzheimer’s disease. Brain. 2022. 10.1093/brain/awac294. 10.1093/brain/awac294 PMC10169283 35949106

[64] Abukar A, Wipplinger M, Hariharan A, Sun S, Ronner M, Sculco M, et al. Double-stranded RNA structural elements holding the key to translational regulation in cancer: the case of editing in RNA-binding motif protein 8A. Cells. 2021;10. 10.3390/cells10123543. 10.3390/cells10123543 PMC8699885 34944051

[65] Nguyen TA, Heng JWJ, Kaewsapsak P, Kok EPL, Stanojević D, Liu H (2022). Direct identification of A-to-I editing sites with nanopore native RNA sequencing. Nat Methods.

[66] Quan Z-J, Li S-A, Yang Z-X, Zhao J-J, Li G-H, Zhang F, et al. GREPore-seq: a robust workflow to detect changes after gene editing through long-range PCR and nanopore sequencing. Genomics Proteomics Bioinformatics. 2022. 10.1016/j.gpb.2022.06.002. 10.1016/j.gpb.2022.06.002 PMC11082256 35752289

[67] Chen L, Ou L, Jing X, Kong Y, Xie B, Zhang N (2023). DeepEdit: single-molecule detection and phasing of A-to-I RNA editing events using nanopore direct RNA sequencing. Genome Biol.

[68] Liu Z, Quinones-Valdez G, Fu T, Huang E, Choudhury M, Reese F (2023). L-GIREMI uncovers RNA editing sites in long-read RNA-seq. Genome Biol.

[69] Robinson E, Tang A. Brooks lab western blotting protocol. protocols.io. 2020. 10.17504/protocols.io.bcsmiwc6. [cited 2020 Mar 20].

[70] Vollmers AC, Mekonen HE, Campos S, Carpenter S, Vollmers C (2021). Generation of an isoform-level transcriptome atlas of macrophage activation. J Biol Chem.

[71] Li H (2016). Minimap and miniasm: fast mapping and de novo assembly for noisy long sequences. Bioinformatics.

[72] Pertea G, Pertea M (2020). GFF utilities: GffRead and GffCompare. F1000Res.

[73] Wick RR (2019). Badread: simulation of error-prone long reads. J Open Source Softw.

[74] Kim D, Paggi JM, Park C, Bennett C, Salzberg SL (2019). Graph-based genome alignment and genotyping with HISAT2 and HISAT-genotype. Nat Biotechnol.

[75] Anders S, Pyl PT, Huber W (2015). HTSeq--a Python framework to work with high-throughput sequencing data. Bioinformatics.

[76] Love MI, Huber W, Anders S (2014). Moderated estimation of fold change and dispersion for RNA-seq data with DESeq2. Genome Biol.

[77] Nowicka M, Robinson MD (2016). DRIMSeq: a Dirichlet-multinomial framework for multivariate count outcomes in genomics. F1000Res.

[78] Reuter JS, Mathews DH (2010). RNAstructure: software for RNA secondary structure prediction and analysis. BMC Bioinformatics.

[79] Long read sequencing of lung adenocarcinoma cell lines. PRJNA981664. BioProject. 2023. https://www.ncbi.nlm.nih.gov/bioproject/?term=PRJNA981664.

[80] Tang A, et al. Long read isoforms with A-to-I edits for H1975 cell line. Zenodo; 2023. 10.5281/zenodo.8019704.

[81] Tang A, Felton C, Brooks A. FLAIR2 results from LRGASP R2C2 sequencing of Human WTC11 and Mouse ES cell lines. Zenodo; 2024. 10.5281/zenodo.11399236.

[82] Tang A, Felton C, Brooks A. FLAIRv2.0. GitHub; 2023. https://github.com/BrooksLabUCSC/flair.

[83] Tang A, Felton C, Brooks A. FLAIR2. Zenodo; 2023. 10.5281/zenodo.11289665.

