# Supplementary material for: Detecting haplotype-specific transcript variation in long reads with FLAIR2
Source: Genome Biol. 2024 Jul 2;25:173. doi: 10.1186/s13059-024-03301-y (PMC11218413; doi:10.1186/s13059-024-03301-y)
Supplement: Supplementary file 5 — Additional file 5. Figures S1-S4. [file 13059_2024_3301_MOESM5_ESM.pdf]

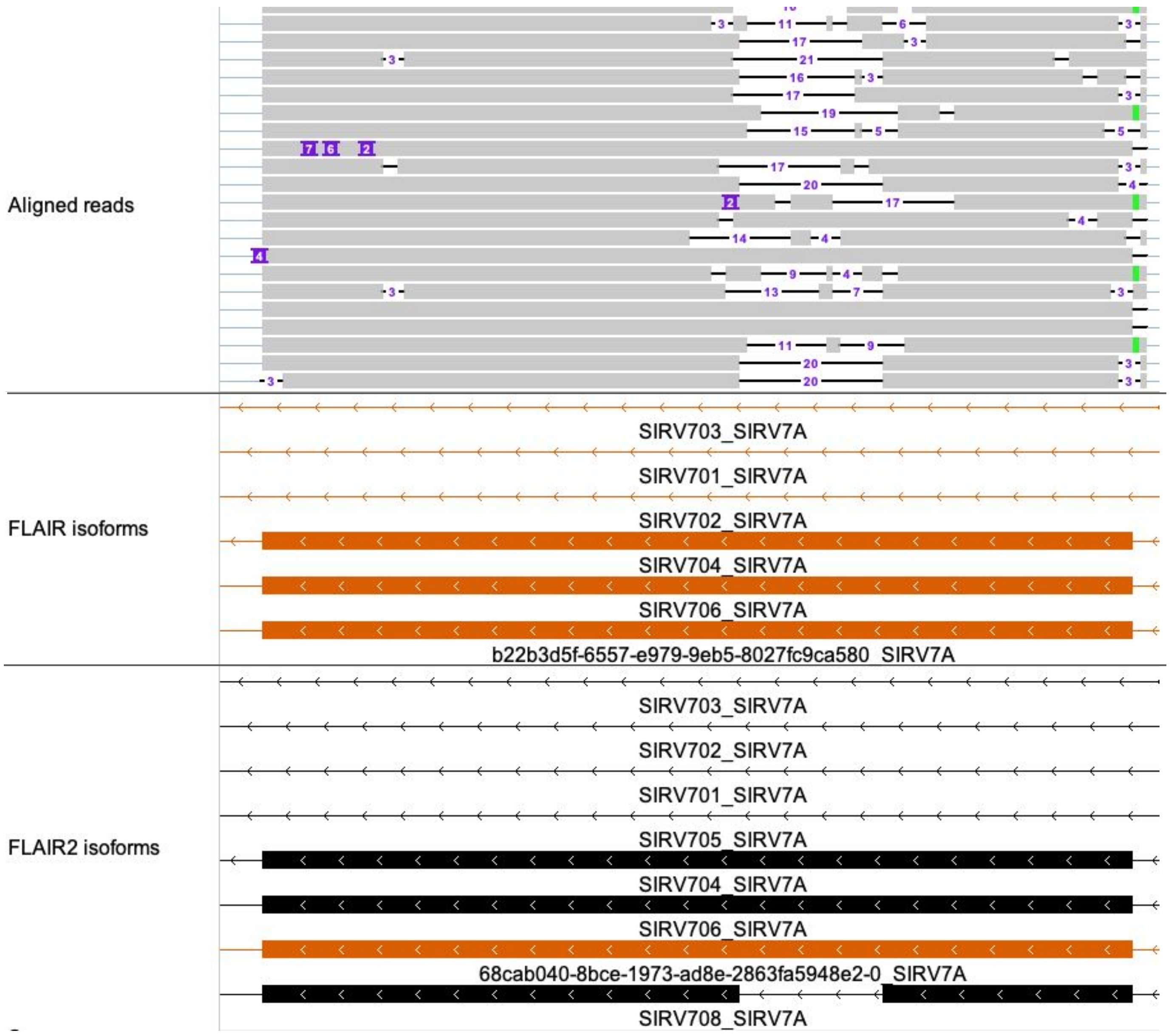

Fig S1 | FLAIR2 improvements on isoform detection. SIRV7A has a small intron which makes it difficult for an alignment tool to map against the genome due to the difficulty of distinguishing and intron from a small deletion. FLAIR2's approach allows for mapping directly to the transcriptome, as a first pass, to identify reads that fully support annotated transcripts.

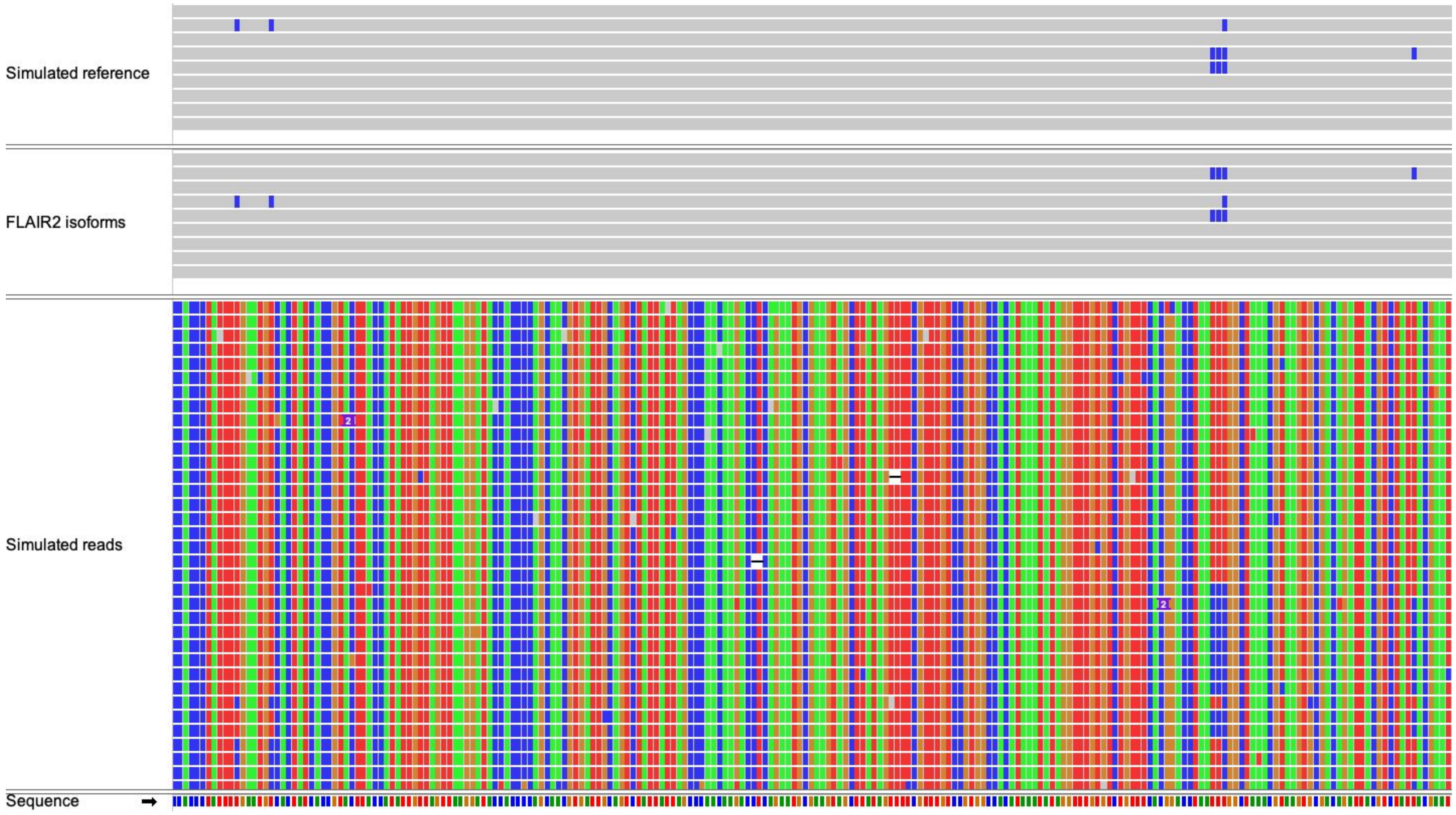

Fig S2 | FLAIR2 has additional capability to identify multiple haplotypes given variant calls. Shown are six simulated variant positions in three distinct patterns. The isoforms and variant combinations that FLAIR2 identifies from simulating reads with fewer than 99% identity. The first track is the reference set of isoforms used for simulation, second track the isoforms identified with FLAIR2, third track the reads that were simulated given different error models displaying all bases, and bottom most track the reference genome sequence.

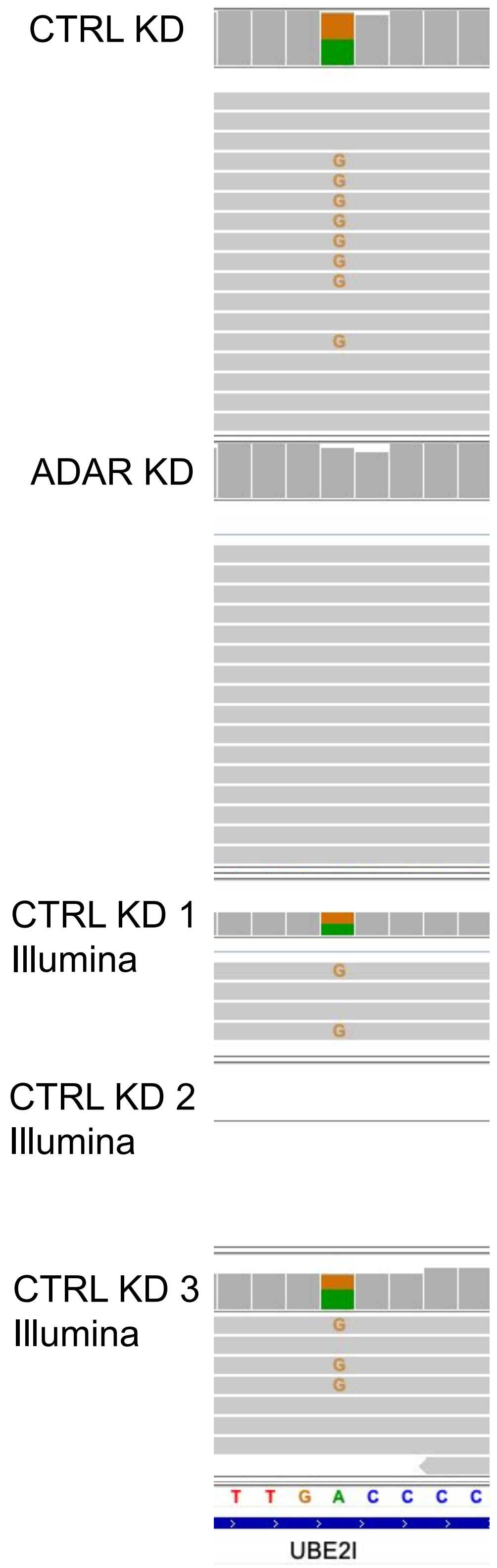

Fig S3 | Example of novel A-to-I editing found with nanopore data. IGV shots of nanopore and Illumina data aligned to hg38. There were no reads aligning to *UBE2I* in the second Illumina CTRL KD replicate.

a

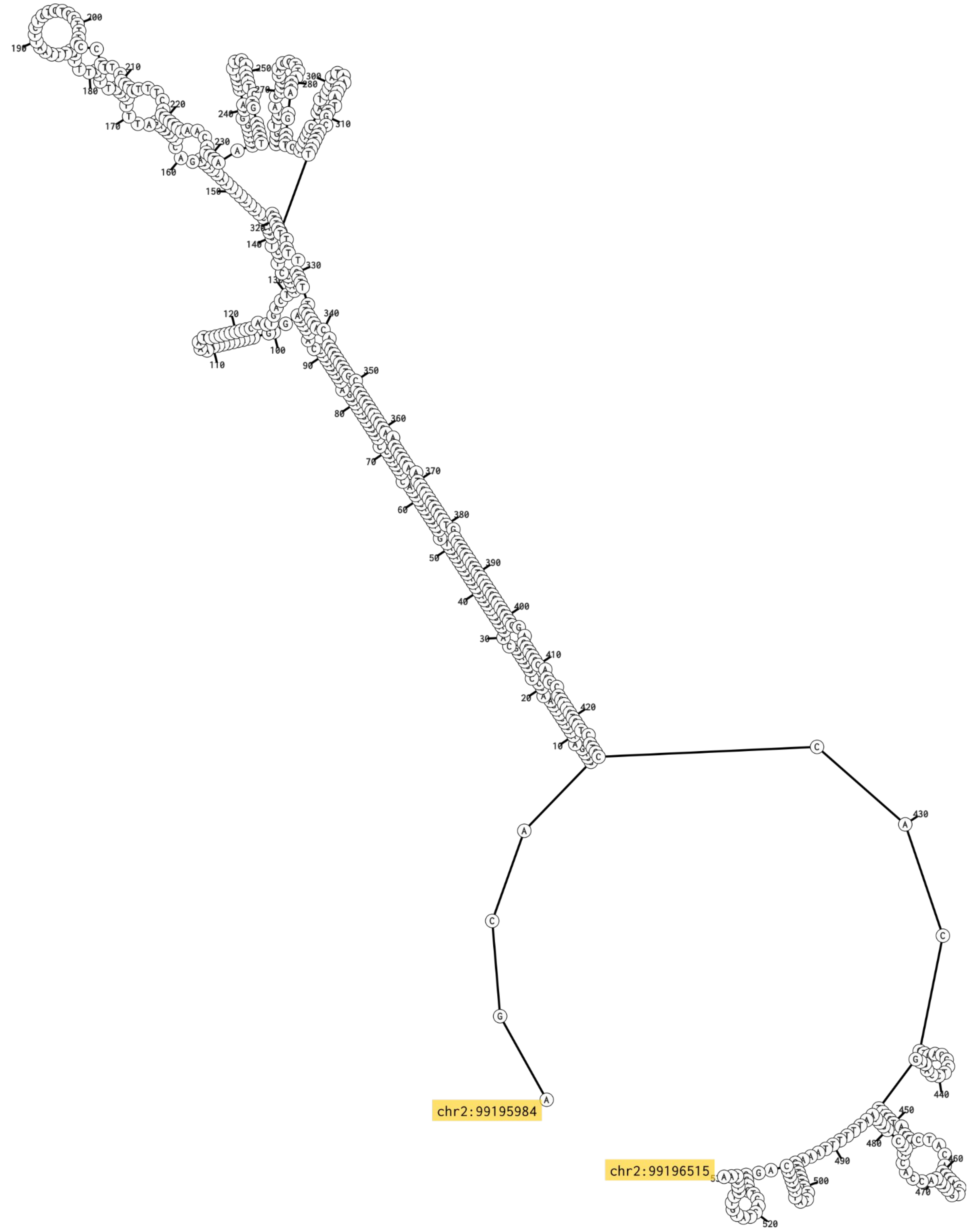

Fig S4 | MRPL30 3’ UTR RNA secondary structure predicted by RNAStructure. The coordinated inosine coordinates are labeled next to the adenosines. Other numeric labels refer to the relative position in the provided sequence for predicting fold structure.
