# Supplementary material for: Detecting haplotype-specific transcript variation in long reads with FLAIR2
Source: Genome Biol. 2024 Jul 2;25:173. doi: 10.1186/s13059-024-03301-y (PMC11218413; doi:10.1186/s13059-024-03301-y)
Supplement: Supplementary file 7 — Additional file 7. Peer review history. [file 13059_2024_3301_MOESM7_ESM.docx]

**Review history**

**First round of review**

**Reviewer 1**

FLAIR2 added a few new features that consider about the RNA variant calls and HSTs. By using short-read and long-read sequencing data of H1975 cells with/without ADAR KD, the authors identified the inosine-isoform associations and their effect on splicing at single-molecular level.
FLAIR2 could be very useful in long read-based research. Below are a few concerns improve the manuscript before publication, including the performance evaluation on different types of long-read sequencing data, such as Oxford Nanopore direct and cDNA sequencing data or Pacbio sequencing data. In this study, the authors evaluated FLAIR2 mostly based on R2C2 data, which is not a widely used long-read sequencing data. Thus, the evaluation on the other types of long-read sequencing data could demonstrate the utility of the tool. A technical suggestion is that the manuscript writing could be improved, since there are some mistakes and unclear descriptions.
Major concerns:
1. In the first part of the result, the authors systematically described the improvements in FLAIR2. It would be good to have a comparison with FLAIR on isoform detection (Table 1) and also a comprehensive performance benchmark with other tools with similar function, such as IsoQuant, Bambu, TALON.
2. How is the performance of FLAIR in detecting HSTs? More comparison can make the conclusion in the second part of the result more reliable.
3. Fig. 2d represents the significantly downregulated inosines events from Illumina and Nanopore R2C2 data. Totally, 131 and 413 sites were identified by Illumina and R2C2 data, respectively, and only 27 overlap sites between them. It is interesting that 68 downregulated editing sites were solely detected with R2C2 but not Illumina. Which means the nanopore sequencing can detect more novel editing sites than Illumina (10 in Fig. 2d). To improve the explanation about this result, I suggest adding the following analysis:
i. If this is caused by coverage, the authors may increase the cutoff to see the overlap of editing sites.
ii. The short-read sequencing should have a higher coverage than Nanopore data, which explains why Illumina can detect more REDIportal database reported editing sites (376 vs. 36) than Nanopore. It's kind of odd that the coverage of some editing sites is higher in Nanopore data. I think it may be due to highly repetitive sequences. The authors can check it out.
iii. It would be also helpful to check 68 novel editing sites and figure out why these editing sites cannot be detected by short-read sequencing.
4. Fig 4d showed a case that editing sites around splicing junctions potentially affect alternative splicing. Are there more cases that have been reported/supported literature?

Minor concenrs:
1. Please add method description of the Illumina data pre-processing, gene expression quantification, and differential expression analysis.
2. In Fig. 2c, what method did the authors use for differential test? The font size of axis and gene symbols is too small to read.
3. In the figure legend of Fig 4a and 4c, 'the top three coverage tracks are Illumina ….'. I think the top two tracks with read alignment should be nanopore while the bottom three coverage tracks are Illumina. Please check the consistency between the figure and their legends.
4. Line 323, please list the barcode sequences on oligo-dTs in a supplementary table.
5. Line 331, what is 'TAE'?
6. Line 334 & 338, what is '65 C' and '4C'?
7. Line 400, what do the authors mean with 'less than 15% putative editing', Is it the ratio of reads with editing on this site?
8. Line 196-197, 'Of these 334 A-to-I events, 312 were downregulated … 12 were upregulated…'. Are there another ten A-to-I events without significant change? Besides, 312 downregulated events were present in the REDIportal database. But I found 403 (376+27) in Fig. 2d. Can the authors clarify these numbers?
9. In Table 3:
a) I am still not clear with the relationships between three Pools and 6 samples (three ADAR KD and three ctrl KD), even when I tried to figure out from the Methods part (Line 319). I think the authors should use a figure to clarify them.
b) What's the difference between the last two rows in the table? They have the same row names.
10. Line 179, I cannot understand the conclusion '70-80% knockdown of ADAR1 protein levels' from Fig. 2b.
11. Line 181, please add the calculation of the ratios '55.1, 73.7, and 78.8% decrease' in the Methods part
12. Line 185, how to calculate the number '9,599 bp' from Table 3?
13. Line 250, it would be informative if the authors show the predicted secondary structure of MRPL30 and label the position of editing sites.

**Reviewer 2**

Tang et al. present FLAIR2, an updated version of the FLAIR algorithm developed by the Brook´s lab, which is one of the most cited tools for Transcript reconstruction from Nanopore reads.
Compared to FLAIR, FLAIR2 is claimed to have improved full-length Transcript identification accuracy and incorporate variant phasing capabilities. The use this new functionality to study patters of A to I editing using a ADAR KD experiment.

Overall, this is a nice work that shows the potential of the R2C2 library preparation and FLAIR2 analysis to investigate haplotype specific transcript expression and A to I editing events. However, the work contains many poorly described or demonstrated analyses which should be carefully revised to improve the quality of the work and of the manuscript.

1. To demonstrate the accuracy of full-length Transcript identification, they use SIRVs and compare their method to StringTie2, FLAMES and FLAIR. The show FLAIR2 has improved precision and comparable sensitivity, meaning that they have a good control of false positives. However, they seem to use the complete SIR1-SIRV7 annotation as reference, therefore their evaluation applies only for known transcripts. Since FLAIR2 includes detection of novel transcripts, a specific evaluation of accuracy for these should also be provided. This could be done by removing a subset of SIRVs and testing the ability of their algorithm to identify those. Another option is that they use SQANTI-SIM, a LRS data simulation tool for novel transcripts. Also, sensitivity values are around 70-80. It would be interesting to know if there are any kind of property in the transcripts that are not being detected, for example, due to low expression, length, or complex splicing pattern.
2. I am curious about the robustness of the method for transcript detection when haplotype variants are present. In the current work, they only show performance on the identification of transcripts without variants -using SIRVs- and then identification of some HTS genes in the mouse sample. Is there an interaction among the two types of detection? For example, how sensitive is the method to the detection of allelic imbalance, where the transcripts from one haplotype are more expressed than those from the other? Also, it is not fully clear that HST refer always to alternative transcript structures (chains of introns) or also only exonic nucleotide variants are included. Detailed information -not just their names- about the 152 genes found to have HTS should be provided as Supplementary table, for including at least the proportions of each haplotype as in 1c (and ideally also as 1b). Moreover, supplementary table 2 is missing. Finally, the section demonstrating HTS shows that FLAIR2 identifies haplotype variants, but not the performance for doing so? How many HTS genes are expected? Some data showing the boundaries for accurate detection of HTS variants should be provided.
3. Please explain how the consensus-called reads described in line 187 is computed.
4. It is unclear if the R2C2 results shown in 2d correspond to consensus detections in all replicates or the pooling of the A to I detection data found in each replicate. As there seem to be a high proportion of A to I changes detected only by FLAIR2 and not with the Illumina sequencing and not present in the REDIportal, the immediate question is if these R2C2 only calls are false positives. What is the consistency in the detection of these novel editing calls?
5. Is the fraction of Type I and Type II hyperedited transcripts found with FLAIR2 the same as what is present in the Illumina and REDIportal data? If not, comment on the differences.
6. In general, methods are not very detailed, and it is not easy to fully understand how things were done:
a. For the variant phasing implementation, longshot and pepper are used. The text is unclear if both methods are integrated in FLAIR2 separately or in combination, or if they have to be run independently. If they are somehow combined, as it seems at least for the variant-caller-agnostic method (line 388), how is this combination performed? Also, Figure 1a shows the FLAIR2 algorithm but this figure is way to simplistic. I encourage to represent better the different steps both for transcript identification and for the allocation of phasing information to isoforms.
b. Please add information about the SIRV1 to SIRV7 data (lengths, exon structures, complexity, etc.). Readers should not have to search for this information.
c. It should be nice to include a supplementary table that lists the differences between FLAIR and FLAIR2 in terms of functionalities and Python programs included.
d. It is unclear how replication was implemented in the siRNA knockdown experiments. Where the three siRNAs (s1007, s1008 and s1009) used once or three times each, where there any replicated for the control experiment? If the experiment was replicated, how were replicates handled? In general, better description of replication for all experimental datasets should be provide.
e. It is unclear how simples were pooled. Each seems like each ADAR KD samples was pooled to a control sample (the same control or different control) to be sequenced together. What is then Pool1 and Pool2 in line 325? Is there any difference between Pool 2(line 325) and pool2 (line 328)? In line 329 it is indicated that Pool1 had 4 samples (which ones?). Are these samples any of the biological replicates described above? All this section of the samples and the pools is very confusing to me. Better description and possibly a figure are needed.
f. In the FLAIR2 algorithm (358), it is indicated that annotated transcripts with sufficient supported reads are put aside, and the remaining reads used for novel isoform discovery. How much support is needed to accept a known transcript? What happens to the reads mapping to annotated transcripts with not enough support? Are they discarded? Are they used for de novo Transcript Discovery? Which % of reads are productive (they end up associated to a Transcript?)
g. How is the collapsing done (364)? Which criteria is applied to create more than one isoform from a group of reads with the same splice junction chain?
h. Line 410 speaks about knockdown condition, does this refer to each of the 3 siRNAs used? Please clarify. How are the different conditions or replicates combined in the Inosine detection in long reads analysis? Please specify the data used to create the contingency table for the Fisher´s exact test used in this section.
i. This inosine coordination analysis also needs more explanation. What method is used to test the concept of coordination? Was this performed genome-wide? Could you elaborate more on the last sentence of this section? How do you require 10 reads to have the edited position splice out?
7. The authors fail to include in the discussion a reference to other attempts to identify A to I editing using nanopore sequencing, which normally uses direct RNA sequencing (e.g. PMID: 35697834, PMID: 35752289, PMID: 37069604) . Some reference to these methods and the potential advantaged of the FLAIR2 approach should be discussed.

**Reviewer 3**

The authors have developed FLAIR2, a tool for isoform detection that incorporates variants called from existing tools, enabling the detection of haplotype-specific transcripts (HSTs). In the manuscript, the authors ran FLAIR2 on a SIRV dataset to benchmark their performance with existing isoform identification tools. With FLAIR2, the authors then performed
1. an analysis on Castaneus x 156 Mouse 129 hybrid mouse embryonic stem cells, demonstrating the ability of the workflow to detect HSTs and identified 158 genes with HSTs and showed they are biologically relevant.
2. an analysis on short and long read cDNA sequencing of samples with and without ADAR1 knockdown, showing the global down-regulation of A-to-I editing following ADAR1 knockdown.

The authors concluded that 1) FLAIR2 improves isoform detection and incorporates sequence variants for haplotype-specific transcript (HST) detection; 2) Using FLAIR2 successfully identified HSTs and HST bias; 3) Long-read sequencing identifies hyperedited transcripts that are missed from short-read sequencing methods for more comprehensive identification of ADAR targets.

Overall, this manuscript addresses the important topic of analysing HSTs and RNA editing. FLAIR2 seems to a very useful tools with various of functionalities and is easy to install and run. The code is also well-written and well-documented. However, some of claims in the manuscript do not seem to be well supported by the results provided.

Major comments:
1. In the manuscript, the authors performed a fairly lightweight benchmark of isoform detection performance and claim that FLAIR2 performs better than other methods. However, the results do not really support this claim.
a) In Table 1: FLAIR2 has similar sensitivity and precision as FLAMES; In Table 2, the results from FLAIR2 and StringTie2 are not comparable due to much higher precision but lower sensitivity. The authors could use a more stringent threshold in StringTie2 to tune down the sensitivity to a comparable level and compare the precision.
b) More recent methods (e.g. bambu, published in Nature Methods) have also shown improved isoform detection performance and should be included in any comparison.
c) SIRV data, with known ground truth, are useful for benchmarking. However, SIRVs are synthetic RNA with a limited number of genes and limited fold-changes. The results on SIRVs may not necessarily be generalisable to real biological RNA datasets. To address this, the authors could also run benchmarking on another biological RNA dataset by holding out a set of known transcripts from the annotation and assessing how many of them can be recovered in the output of each method.
The authors do not necessarily have to show that FLAIR2 outperforms all the existing isoform identification tools regarding overall performance. FLAIR2 includes transcript mapping, which could theoretically improve small or micro-exon detection. In addition, FLAIR/FLAIR2 could use the short reads to refine the splice junction, while the other benchmarked methods do not. The authors could potentially assess the performance of micro-exon and splice junction to highlight the benefits of using FLAIR2 for isoform detection. Besides, the potential of FLAIR2 to categorise haplotype-specific transcripts (HSTs) could still benefit the field - most other tools do not provide this option.

2. In the section "Assessing FLAIR2 for haplotype-specific transcript detection", the authors identified 152 genes that contain HSTs and have shown some biological relevance of these genes. However, to highlight the novelty, it might be better to show that most of the genes can not be identified without using long reads. To address this, the authors could do a similar analysis that uses short reads and highlight that the number of HSTs or genes with HSTs are significantly lower without long reads. Many haplotype specific analyses can be performed using short read data, but obviously fewer reads will be informative as they most will not include a relevant variant.

3. In the section "Inosine detection in short and long reads", the authors contrast the significantly changed A-to-I events between samples with and without ADAR knockdown and claimed that long-read sequencing identifies transcripts that are missed from short-read sequencing methods, hence are advantageous for detecting certain novel A-to-I events. However, the overlap between the identification from long and short reads is very small, raising the concern that the additional events identified from the long reads might not be accurate. The authors might consider including more analysis to show that the "novel" events are not extensively false positives.
a) Could the authors please report the total number of A-to-I edits identified, out of which the significantly changed A-to-I events were identified? If the total number of A-to-I edits identified is relatively large, it might be helpful to do multiple testing corrections to prevent an extensive number of false discoveries. Otherwise, the authors should comment on why the false discoveries are not concerning in downstream analysis.
b) It seems most of the A-to-I events, if significantly changed after ADAR knockout, are more expected to be down-regulated. Could the authors please report the number of significantly up-regulated events to see whether an unexpected number is observed?
c) Over half (68/131) of down-regulated A-to-I events are on novel A-to-I edit sites, which were not supported by short reads or in REDIportal. Given that these events were also considered when the authors identified "type II hyperediting" downstream, could the authors please explain why they are not identified by short reads if they are real? To address this the authors could include some statistics e.g., how many of the 68 events are from identified A-to-I sites (not necessarily downregulated) from short reads and their p-values. The idea is to verify the existence of any signal, even if it is weak, in short reads that could verify the novel events.

4. Some presentation of the results are unclear and hard to understand. For instance, the numbers in the text seem not to be consistent with the results shown in Figure 2d. Figure 2d shows 413 significantly down-regulated A-to-I events, while the number is 312 at line 196. The authors should either revise the number or explain the inconsistency. Based on Figure 2d, the authors identified 63 significantly down-regulated A-to-I events from long reads overlapped with REDIportal. However, in lines 203-206, the authors stated that 63 significantly changed long-read A-to-I events overlapped with REDIportal, and 62 were down-regulated.

Minor comments
1. For reproducibility, it's better to provide the scripts used in all analyses, including the commands used when running FLAIR2. Particularly, it is not clear whether the short-read junctions were used in each FLAIR2 run.
2. For SIRV analysis, it is not clear which SIRV annotation is provided. I believe there are different versions of annotation, including a complete annotation, an incomplete annotation and an over-annotated annotation.
3. In the FLAIR2 software GitHub page, it might be better to provide an example of data and code incorporating the longshot or PEPPER-margin-Deepvariant, and document the expected output.
4. At line 161, when validating the 152 genes identified to contain HST bias, would it be possible to report the total number of known imprinting genes (perhaps after removing lowly expressed ones)? This might provide some idea of the sensitivity of the HST identification.
5. In Table 3, it is not super clear why the number of size-selected reads is only for pool 3. It is also not clear what pool 3 is as it was not mentioned in the text. In the paragraph, starting at line 171, all the description was per-replicate instead of per-pool. It could be clearer if the authors also summarise the sequencing per replicate.
6. Line 202: could the authors please report the number of variants called by each variant caller and their overlaps to show the consistency of these variant callers?
7. Line 215: It is not clear how much difference in the coverage between short reads and long reads. The authors could potentially report the average coverage.
8. In the discussion, could the authors please comment on the potential usage of FLAIR2 on non-R2C2 data?

**Authors’ response to reviewers**

We would like to thank the reviewers for the positive and constructive feedback.

A consistent major concern by all three reviewers was the lack of a comprehensive benchmark on the performance of FLAIR2 compared with other tools and using other datasets other than R2C2. We would like to note that this is a coordinated submission with the Long-read RNA-Seq Genome Annotation Assessment Project (LRGASP) Consortium comprehensive and systematic evaluation of long-read transcriptome analysis methods. The version of FLAIR2 presented in this manuscript is the version used for the LRGASP evaluation. The bioRxiv detailing the LRGASP evaluation was released after submission of this manuscript. The bioRxiv can now be found at this link: <https://www.biorxiv.org/content/10.1101/2023.07.25.550582v1>. The paper was submitted to Nature Methods and it has been accepted-in-principle. FLAIR(v2) was identified as one of the top performing tools for both annotated and novel transcript detection based on a more extensive assessment which included, real data from multiple library preps and sequencing platforms (e.g. cDNA, direct RNA, Oxford Nanopore and PacBio), simulated data, spike-in RNA variants, and manual curation; therefore, we did not include our own benchmark in this paper. Moreover, FLAIR is mentioned as one of the tools for for transcript detection of both annotated and novel transcripts in the LRGASP Consortium’s Final Recommendations. Since the bioRxiv of the LRGASP results are now available, we have referenced it in this revised manuscript.

For the LRGASP study, we did not submit results for R2C2 libraries as we had not had extensive experience running FLAIR with R2C2 libraries at the time. It is appropriate to now include this evaluation in this submitted manuscript, since the data produced here was with the R2C2 protocol. We now include evaluation results of FLAIR2 on LRGASP-sequenced R2C2 SIRV cDNA and compare the FLAIR2 results with Bambu, IsoQuant, Lyric, Mandalorion, and TALONG LRGASP submissions using the same LRGASP metrics and publicly available LRGASP scripts to perform the evaluation.

Here is an excerpt of the resulting evaluation, which is now included in our revised manuscript: *This set of SIRVs contain “spliced SIRVs” which are synthetic transcripts with alternative splicing patterns to mimic human gene RNA processing complexity. The “unspliced SIRVs” were a set of synthetic transcripts with no shared alternative splicing patterns, but were very long transcripts (4-12kb). For spliced SIRVs, FLAIR was among the top performing tools in both sensitivity and precision; however, on long SIRVs FLAIR was among the worst performing tools whereas Bambu and IsoQuant had the highest sensitivity and precision (Supplementary Table 2). As noted in the LRGASP assessment (Francisco J. Pardo-Palacios et al. 2023), different tools will weight the annotation differently when calling isoforms, which explains the poor performance of FLAIR on long SIRVs. Most library preparation methods resulted in a majority of sequences less than 4kb; therefore, there were few reads that fully sequenced a long SIRV from*

*end-to-end(Francisco J. Pardo-Palacios et al. 2023). Even in the annotation-reliant mode, FLAIR will only determine if an annotated isoform is present if there is read-level evidence supporting the entire isoform, while other tools may accept partial support.*

*Giving less weight to annotated transcripts allows for better novel isoform detection.*

In summary, FLAIR2 has been extensively evaluated through the LRGASP Consortium and we include additional evaluation on SIRV sequenced with the R2C2 approach. FLAIR2 has been found to be one of the top performing tool for both annotated and novel transcript detection on a variety of metrics.

Our point-by-point responses for additional reviewer comments now follows. Reviewer reports:

**Reviewer #1:** FLAIR2 added a few new features that consider about the RNA variant calls and HSTs. By using short-read and long-read sequencing data of H1975 cells with/without ADAR KD, the authors identified the inosine-isoform associations and their effect on splicing at

single-molecular level.

FLAIR2 could be very useful in long read-based research. Below are a few concerns improve the manuscript before publication, including the performance evaluation on different types of long-read sequencing data, such as Oxford Nanopore direct and cDNA sequencing data or Pacbio sequencing data. In this study, the authors evaluated FLAIR2 mostly based on R2C2 data, which is not a widely used long-read sequencing data. Thus, the evaluation on the other types of long-read sequencing data could demonstrate the utility of the tool. A technical suggestion is that the manuscript writing could be improved, since there are some mistakes and unclear descriptions.

Major concerns:

1. In the first part of the result, the authors systematically described the improvements in FLAIR2. It would be good to have a comparison with FLAIR on isoform detection (Table 1) and also a comprehensive performance benchmark with other tools with similar function, such as IsoQuant, Bambu, TALON.

As mentioned in the high-level response, we did not perform extensive benchmarking for FLAIR2 in this paper as it was already included in the LRGASP paper, the timing of which was such that the bioRxiv results were posted after our submission. In the LRGASP evaluation, FLAIR was determined to be one of the top performing tools. IsoQuant and Bambu also performed very well. The main difference is that IsoQuant and Bambu more heavily weights annotations and allows for partial read support, while FLAIR requires complete read support in transcript detection. This allows for better novel isoform detection, which was noted as a strength of FLAIR in the LRGASP paper.

2. How is the performance of FLAIR in detecting HSTs? More comparison can make the conclusion in the second part of the result more reliable.

With moderately accurate data, we find that FLAIR2 can recover HSTs based on a simulated study. We simulated transcripts with an error rate of 99% (similar to the R2C2 reads median 99.3% accuracy) with a manually created, complex haplotype structure made to mimic A-to-I editing patterns. FLAIR2 was able to incorporate 15/15 variants correctly when using FLAIR2’s

variant assigning method. This is now included in the paper and a new supplemental figure (Supplementary Figure 2). Users of FLAIR have the option to perform their own phasing, such as with longshot which is currently capable of only of diploid phasing, or FLAIR2’s method which can create as many haplotypes that have sufficient read support. This simulation demonstrates how FLAIR2 performs with complex variant data.

3. Fig. 2d represents the significantly downregulated inosines events from Illumina and Nanopore R2C2 data. Totally, 131 and 413 sites were identified by Illumina and R2C2 data, respectively, and only 27 overlap sites between them. It is interesting that 68 downregulated editing sites were solely detected with R2C2 but not Illumina. Which means the nanopore sequencing can detect more novel editing sites than Illumina (10 in Fig. 2d). To improve the explanation about this result, I suggest adding the following analysis:
   i. If this is caused by coverage, the authors may increase the cutoff to see the overlap of editing sites.
   ii. The short-read sequencing should have a higher coverage than Nanopore data, which explains why Illumina can detect more REDIportal database reported editing sites (376 vs. 36) than Nanopore. It's kind of odd that the coverage of some editing sites is higher in Nanopore data. I think it may be due to highly repetitive sequences. The authors can check it out.
   iii. It would be also helpful to check 68 novel editing sites and figure out why these editing sites cannot be detected by short-read sequencing.
   iv. Fig 4d showed a case that editing sites around splicing junctions potentially affect alternative splicing. Are there more cases that have been reported/supported literature?

Between Illumina and R2C2, the coverage difference is large and we have already implemented minimum coverage and significance filters for R2C2 inosine detection so we did not decrease the coverage minimum there. It is true that doing so would increase the number of inosines detected in R2C2 and likely increase the proportion of those inosines that overlap Illumina/REDIportal. There was only mild enrichment of repetitive regions around the A-to-Is that were missed by Illumina – those sites were more 6.8% likely to be in repetitive regions. For the editing sites detected only in R2C2, shown to be knocked down by A-to-I, and found in REDIportal and not Illumina, we have examined coverage differences and observed that those sites were more likely to be in repetitive regions and that Illumina read coverage was lower. We have added this analysis to the main text now. There are other cases in the literature of splicing disruptions due to inosines, which we have now included e.g. [SJ Tang et al.](https://www.nature.com/articles/s41467-020-14621-5)

Minor concerns:

1. Please add method description of the Illumina data pre-processing, gene expression quantification, and differential expression analysis.

We have added the Illumina data processing to the methods, thank you. We did not use any custom Illumina tools and users should be able to follow the analyses readily.

2. In Fig. 2c, what method did the authors use for differential test? The font size of axis and gene symbols is too small to read.

Figure 2c, we used DESeq2 which are based on a negative binomial generalized linear model. This was not previously mentioned in the paper and has been added to the methods section. We have increased the text size for this panel.

3. In the figure legend of Fig 4a and 4c, 'the top three coverage tracks are Illumina ….'. I think the top two tracks with read alignment should be nanopore while the bottom three coverage tracks are Illumina. Please check the consistency between the figure and their legends.

Thank you, this is a typo and we have fixed the figure legends for 4a/c.

4. Line 323, please list the barcode sequences on oligo-dTs in a supplementary table.

We have included a supplementary table 6 with the oligo-dTs indexes now and also included the URL to the full splint sequences and the protocol to generate the splints in the methods section now.

5. Line 331, what is 'TAE'?

We have added this into the methods now, TAE is a buffer made with Tris, acetate, and EDTA.

6. Line 334 & 338, what is '65 C' and '4C'?

Here we have added a degree symbol to clarify these are incubation temperatures.

7. Line 400, what do the authors mean with 'less than 15% putative editing', Is it the ratio of reads with editing on this site?

Yes this is the ratio of reads that are edited, if too few reads were edited then it was pre-filtered from our analysis to identify A-to-I edits. We have clarified the sentence better: “Positions were considered putatively edited if the SNV was G→A or T→C, and positions with less than 15% of reads representing the edited base were filtered out.”

8. Line 196-197, 'Of these 334 A-to-I events, 312 were downregulated … 12 were upregulated…'. Are there another ten A-to-I events without significant change? Besides, 312 downregulated events were present in the REDIportal database. But I found 403 (376+27) in Fig. 2d. Can the authors clarify these numbers?

We have fixed the numbers in the text to match with the results in Fig 2d and supplementary table 3. It is now “We identified 413 A→G mismatches in the Illumina data that were significantly changed upon ADAR knockdown (Methods), with the majority (403) of these positions present in the REDIportal database. Of the A-to-I events identified with short reads, 409 were downregulated in the knockdown conditions and 4 were upregulated.”

9. In Table 3:
   a) I am still not clear with the relationships between three Pools and 6 samples (three ADAR KD and three ctrl KD), even when I tried to figure out from the Methods part (Line 319). I think the authors should use a figure to clarify them.
   b) What's the difference between the last two rows in the table? They have the same row names.

For point A, we did not add a figure but have added clarifying sentences. This added sentence should help improve the text more: “On the first flow cell, the ADAR KD replicate #1 and CTRL KD replicate #1 were pooled and sequenced together as Pool 1, and so on for the other two replicates for each knockdown condition and flow cells.” We also removed confusing wording and added more detail about the size selection steps which only apply to Pool 1.

For point B, we fixed the row names, thank you. One was for the size selected ADAR KD replicate #1 and the other row was for the size selected CTRL KD replicate #1.

10. Line 179, I cannot understand the conclusion '70-80% knockdown of ADAR1 protein levels' from Fig. 2b.

We have added this to the methods now too: “Relative protein abundance levels were measured from blot images using FIJI software to calculate degree of knockdown with ACTB as the loading controls to normalize.”

11. Line 181, please add the calculation of the ratios '55.1, 73.7, and 78.8% decrease' in the Methods part

We have added it to the Methods now: “The level of ADAR knockdown in each replicate was calculated comparing the normalized level of ADAR expression in short reads in each control knockdown replicate with its corresponding ADAR knockdown replicate (same-numbered replicate).”

12. Line 185, how to calculate the number '9,599 bp' from Table 3?

We have now clarified in the text that 9,599 is the median raw read length. It is not what was written in Table 3 and have removed confusion to the reference to table 3. In table 3 we have the total number of bases sequenced for the raw reads. Consensus called reads reduce the median read length to 1,287, indicating that the original circularized molecules are sequenced around 7 times. Table 3 is now Table 1 in our revised manuscript.

13. Line 250, it would be informative if the authors show the predicted secondary structure of MRPL30 and label the position of editing sites.

We have added the predicted structure of the 3’ UTR hairpin for MRPL30 including the two coordinated inosines, Supplementary Figure 4.

**Reviewer #2:** Tang et al. present FLAIR2, an updated version of the FLAIR algorithm developed by the Brook´s lab, which is one of the most cited tools for Transcript reconstruction from Nanopore reads.

Compared to FLAIR, FLAIR2 is claimed to have improved full-length Transcript identification accuracy and incorporate variant phasing capabilities. The use this new functionality to study patters of A to I editing using a ADAR KD experiment.

Overall, this is a nice work that shows the potential of the R2C2 library preparation and FLAIR2 analysis to investigate haplotype specific transcript expression and A to I editing events.

However, the work contains many poorly described or demonstrated analyses which should be carefully revised to improve the quality of the work and of the manuscript.

1. To demonstrate the accuracy of full-length Transcript identification, they use SIRVs and compare their method to StringTie2, FLAMES and FLAIR. The show FLAIR2 has improved precision and comparable sensitivity, meaning that they have a good control of false positives. However, they seem to use the complete SIR1-SIRV7 annotation as reference, therefore their evaluation applies only for known transcripts. Since FLAIR2 includes detection of novel transcripts, a specific evaluation of accuracy for these should also be provided. This could be done by removing a subset of SIRVs and testing the ability of their algorithm to identify those. Another option is that they use SQANTI-SIM, a LRS data simulation tool for novel transcripts. Also, sensitivity values are around 70-80. It would be interesting to know if there are any kind of property in the transcripts that are not being detected, for example, due to low expression, length, or complex splicing pattern.

As mentioned at the beginning of our response, a more comprehensive evaluation of FLAIR2 was included in the LRGASP evaluation. From the LRGASP work, FLAIR2 novel transcript detection was evaluated using real data, simulated data, and GENCODE manual creation.

FLAIR2 is reported to be the recommended tool for novel detection by the LRGASP Consortium.

The consortium also examined some of the long SIRV transcripts that were not able to be detected, because the transcripts were not represented in full in the sequencing data. FLAIR2 is not capable of scaffolding long reads to call transcripts, and instead only calls transcripts that are supported by x reads end-to-end, where x is a specified read coverage threshold. Other long read tools either scaffold multipel reads or rely more heavily on the annotation and report annotated transcripts that have only partial read support.

2. I am curious about the robustness of the method for transcript detection when haplotype variants are present. In the current work, they only show performance on the identification of transcripts without variants -using SIRVs- and then identification of some HTS genes in the mouse sample. Is there an interaction among the two types of detection? For example, how sensitive is the method to the detection of allelic imbalance, where the transcripts from one haplotype are more expressed than those from the other? Also, it is not fully clear that HST refer always to alternative transcript structures (chains of introns) or also only exonic nucleotide variants are included. Detailed information -not just their names- about the 152 genes found to have HTS should be provided as Supplementary table, for including at least the proportions of each haplotype as in 1c (and ideally also as 1b). Moreover, supplementary table 2 is missing. Finally, the section demonstrating HTS shows that

FLAIR2 identifies haplotype variants, but not the performance for doing so? How many HTS genes are expected? Some data showing the boundaries for accurate detection of HTS variants should be provided.

The identification of isoform structures and of variants are two independent steps, which we have now added clarifying text for. FLAIR currently is not a variant caller, and can only incorporate variants if the tool used for variant calling is capable of detecting haplotypes. One method of running FLAIR is to provide diploid phased variants that are called by longshot. The other method of running FLAIR is to provide variants called by another tool (longshot or other), and FLAIR’s variant assignment method will simply identify all the different combinations of variants observed in the read-level data and any combinations that surpass 3 reads (can be toggled) for an isoform will be output. This is also now better described in the text. This latter method can detect alleles in haplotypes with much less frequency than other alleles, so long as there is sufficient read support, but as such is not limited to only two alleles. This is important for cases of A-to-I editing.

We have added the supplementary table of GO terms that was been missing (now Supplementary Table 3).

Lastly using simulated reads with similar accuracy to our R2C2 libraries, FLAIR2 can recover HSTs with its assignment method. We simulated transcripts with a manually created, complex haplotype structure made to mimic A-to-I editing patterns. FLAIR2 was able to incorporate 15/15 variants correctly (now Supplemental Figure 2). This simulation demonstrates how FLAIR2 performs with complex data and moderately error-prone reads. We have also provided a supplementary data file 4 that contains the variant-isoform associations and their pvalues for the mouse HST analysis.

3. Please explain how the consensus-called reads described in line 187 is computed. The method was developed in the Vollmers lab and we have added a citation to the paper to describe the C3POa software. We have added more detail to the methods i.e.. “From the basecalled raw reads that contain multiple passes over the original circularized read, C3POa identifies and combines repeats, or subreads [[62]](https://paperpile.com/c/UBnig6/Dcxt).“
4. It is unclear if the R2C2 results shown in 2d correspond to consensus detections in all replicates or the pooling of the A to I detection data found in each replicate. As there seem to be a high proportion of A to I changes detected only by FLAIR2 and not with the Illumina sequencing and not present in the REDIportal, the immediate question is if these R2C2 only calls are false positives. What is the consistency in the detection of these novel editing calls? We manually reviewed novel R2C2 events’ read level data in IGV and additionally only considered events where we observed a decrease in editing upon ADAR knockdown. We now have included in the text a noted average decrease in Illumina coverage in these regions, as well as from manual review as shown in Fig 4c and 4d from poor coverage of the edit. Without additional validation, these novel positions can still be false positives despite our efforts to vet them. We have clarified in the text that these novel positions, while we try to implement filters, can still be false positives.
5. Is the fraction of Type I and Type II hyperedited transcripts found with FLAIR2 the same as what is present in the Illumina and REDIportal data? If not, comment on the differences.

Type II editing is difficult to identify with short reads due to challenges in alignment with too many mismatches. As a result, Type II editing may be underrepresented in Illumina/REDIportal data. There is no gold standard benchmark so differences in editing distributions are difficult to quantify between short/long reads. We would require more data to validate this, so for the scope of this publication we have not done this comparison.

6. In general, methods are not very detailed, and it is not easy to fully understand how things were done:
   a. For the variant phasing implementation, longshot and pepper are used. The text is unclear if both methods are integrated in FLAIR2 separately or in combination, or if they have to be run independently. If they are somehow combined, as it seems at least for the

variant-caller-agnostic method (line 388), how is this combination performed? Also, Figure 1a shows the FLAIR2 algorithm but this figure is way to simplistic. I encourage to represent better the different steps both for transcript identification and for the allocation of phasing information to isoforms.

We have added more details of our integration of longshot and PEPPER-Margin-DeepVariant – “Of the variants that overlapped with REDIportal, longshot identified 1,230 variants and PEPPER-Margin-DeepVariant identified 3,502. We combined all the variant calls from both tools for increased sensitivity, the union resulting in 4,020 putative A-to-I events.” Default parameters of P-M-DV are more sensitive and there is an overlap of 712 variants that are putative A-to-Is called. FLAIR is not a variant caller so we did not evaluate which caller is more accurate and instead took the union and did other filtering to determine A-to-Is. We have also provided more detail on how to integrate variants into FLAIR isoforms: “phasing information from longshot [...] is comprised of a phase set determined for each read and a set of variants corresponding to each phase set. FLAIR2 checks whether multiple reads that are assigned to the same isoform are also assigned by longshot to the same phase set. If these conditions are met with sufficient support for an isoform and phase set, then all variants belonging to that phase set will be associated with that isoform.”

b. Please add information about the SIRV1 to SIRV7 data (lengths, exon structures, complexity, etc.). Readers should not have to search for this information.

We have added a link to the Lexon SIRV Set 4 that was used in our study. In the methods we added more information on the SIRVs, “These are 69 synthetic RNA transcripts on the SIRV1-7 chromosomes made to mimic a complex human transcriptome with genes that are alternatively spliced. Each transcript has on average 5.17 exons with 316 unique exons shared between these transcripts.” Additional information on SIRV benchmarking can be found in the LRGASP paper.

c. It should be nice to include a supplementary table that lists the differences between FLAIR and FLAIR2 in terms of functionalities and Python programs included.

We did not create a table with the additional functionality of FLAIR2, mainly because FLAIR2’s major improvement is contained to the collapse step. Now the methods section includes these sentences for clarity:

“While new options for users have been added, the *align* and *correct* module core algorithms have not changed. [...] FLAIR2’s updates to isoform detection from the previous version are the splice site fidelity checking and annotation reliant additional alignment that can be specified in the *collapse* module.“ We also highlight the new functionalities of FLAIR2 in our online documentation: https://flair.readthedocs.io/en/latest/flair2_functions.html

d. It is unclear how replication was implemented in the siRNA knockdown experiments. Where the three siRNAs (s1007, s1008 and s1009) used once or three times each, where there any replicated for the control experiment? If the experiment was replicated, how were replicates handled? In general, better description of replication for all experimental datasets should be provide.

We have added a sentence to the main text to hopefully clarify that there are 3 biological replicates of ADAR knockdown (each replicate is a different ADAR siRNA) and 3 technical replicates of a control siRNA knockdown (each replicate received the same siRNA). These 6 samples are sequenced on 3 flow cells, which we have also clarified further in the methods. “First, ADAR1 knockdown was performed in H1975 cells using three different ADAR1 siRNAs (see Methods) to achieve 70-80% knockdown of ADAR1 protein levels compared to control replicates (Fig 2b). We also used a control siRNA to perform a knockdown, generating three technical replicates. Next, we sequenced the cDNA from three ADAR knockdown and three control knockdown samples. “

e. It is unclear how simples were pooled. Each seems like each ADAR KD samples was pooled to a control sample (the same control or different control) to be sequenced together. What is then Pool1 and Pool2 in line 325? Is there any difference between Pool 2(line 325) and pool2 (line 328)? In line 329 it is indicated that Pool1 had 4 samples (which ones?). Are these samples any of the biological replicates described above? All this section of the samples and the pools is very confusing to me. Better description and possibly a figure are needed.

Thank you for the close reading of the pools and we now have edited the main text to be more careful with the use of the confusing words “sample” and “pool”:

*To reduce nanopore flow cell variability batch effects, each ADAR KD sample was pooled with a control KD sample for a total of three pools of six barcoded samples. Each pool was sequenced on a separate flow cell*

Most critical is clarifying that each flow cell has 1 ADAR KD replicate and 1 CTRL replicate that are sequenced together as a pool to prevent batch effects. So pool 1 contains ADAR KD replicate #1 and CTRL KD replicate #1, pool 2 contains the second replicate of each condition, and pool 3 the final replicate of each knockdown condition. We have made a separate section in the methods to describe the size selection to increase consensus accuracy, which applies to pool 3.

f. In the FLAIR2 algorithm (358), it is indicated that annotated transcripts with sufficient supported reads are put aside, and the remaining reads used for novel isoform discovery. How much support is needed to accept a known transcript? What happens to the reads mapping to annotated transcripts with not enough support? Are they discarded? Are they used for de novo Transcript Discovery? Which % of reads are productive (they end up associated to a Transcript?)

By default and for our runs of FLAIR2, three supporting reads are necessary to accept a known transcript, although this can be toggled by the user. Read support stringency can be enforced with the –stringent and –check_splice parameters, which we used (as shown in the

now-included flair collapse command). These enforce that the supporting read have bases that align to all the splice junctions for the known transcript and cover the majority of the transcript’s bases including at the ends.

If a read supports an annotated transcript but that annotated transcript does not meet the minimum threshold for an annotated transcript to be included in the final set, then that read is used for de novo discovery.

For our ADAR data set, 67% of reads are “productive” and assigned to an isoform. The percentage of useful reads could change depending on the quality of the sequencing data and the stringency of isoform calling.

g. How is the collapsing done (364)? Which criteria is applied to create more than one isoform from a group of reads with the same splice junction chain?

We have added to the methods the criteria for the collapse TSS determination. These collapsing parameters are the default for FLAIR–a maximum of 2 TSSs and TESs are picked per splice junction chain, and these starts and ends are picked based on those that are most frequent within a fuzzy window calculated for each potential end.

h. Line 410 speaks about knockdown condition, does this refer to each of the 3 siRNAs used? Please clarify. How are the different conditions or replicates combined in the Inosine detection in long reads analysis? Please specify the data used to create the contingency table for the Fisher´s exact test used in this section

We have worded this text better now. “Next, we combined counts of either allele from the control knockdown replicates together and the ADAR knockdown replicates together. [...] We performed a Fisher’s exact test using the number of unedited and edited reads in the ADAR knockdown or control knockdown to assess the significance of the A-to-I differences. ”

i. This inosine coordination analysis also needs more explanation. What method is used to test the concept of coordination? Was this performed genome-wide? Could you elaborate more on the last sentence of this section? How do you require 10 reads to have the edited position splice out?

We have added more clarity to the inosine-inosine coordination analysis in the methods now. “For the long-range inosine coordination analysis to test for inosines that were more frequently

edited together, we first considered inosines that were at least 50 bp apart. The number of edited and unedited reads at each position was assessed for significance with a Fisher’s exact test and this was repeated for all pairs of inosines that appeared on the same molecules.”

For the inosine-intron analysis, for each inosine position, we filtered for positions that had at least 10 reads that were edited and 10 reads where that read had spliced out that position. They are mutually exclusive in this case, with some reads covering the position either with or without edit, and some reads that skip the position. We have clarified this by not calling the position “edited” when a given read has spliced the position out.

7. The authors fail to include in the discussion a reference to other attempts to identify A to I editing using nanopore sequencing, which normally uses direct RNA sequencing (e.g. PMID: 35697834, PMID: 35752289, PMID: 37069604) . Some reference to these methods and the potential advantaged of the FLAIR2 approach should be discussed.

We have now included a discussion of identification of A-to-I editing using nanopore and PacBio sequencing with the above references in our discussion.

**Reviewer #3:** The authors have developed FLAIR2, a tool for isoform detection that incorporates variants called from existing tools, enabling the detection of haplotype-specific transcripts (HSTs). In the manuscript, the authors ran FLAIR2 on a SIRV dataset to benchmark their performance with existing isoform identification tools. With FLAIR2, the authors then performed

1. an analysis on Castaneus x 156 Mouse 129 hybrid mouse embryonic stem cells, demonstrating the ability of the workflow to detect HSTs and identified 158 genes with HSTs and showed they are biologically relevant.
2. an analysis on short and long read cDNA sequencing of samples with and without ADAR1 knockdown, showing the global down-regulation of A-to-I editing following ADAR1 knockdown.

The authors concluded that 1) FLAIR2 improves isoform detection and incorporates sequence variants for haplotype-specific transcript (HST) detection; 2) Using FLAIR2 successfully identified HSTs and HST bias; 3) Long-read sequencing identifies hyperedited transcripts that are missed from short-read sequencing methods for more comprehensive identification of ADAR targets.

Overall, this manuscript addresses the important topic of analysing HSTs and RNA editing. FLAIR2 seems to a very useful tools with various of functionalities and is easy to install and run. The code is also well-written and well-documented. However, some of claims in the manuscript do not seem to be well supported by the results provided.

Major comments:

1. In the manuscript, the authors performed a fairly lightweight benchmark of isoform detection performance and claim that FLAIR2 performs better than other methods. However, the results do not really support this claim.
   a) In Table 1: FLAIR2 has similar sensitivity and precision as FLAMES; In Table 2, the results from FLAIR2 and StringTie2 are not comparable due to much higher precision but lower sensitivity. The authors could use a more stringent threshold in StringTie2 to tune down the sensitivity to a comparable level and compare the precision.
   b) More recent methods (e.g. bambu, published in Nature Methods) have also shown improved isoform detection performance and should be included in any comparison.

As discussed in the beginning of our response, there is now a more comprehensive evaluation of FLAIR2 against other methods with multiple benchmarks through the LRGASP Consortium. We now have added to the text FLAIR2’s performance and comparison with other tools via the LRGASP consortium.

c) SIRV data, with known ground truth, are useful for benchmarking. However, SIRVs are synthetic RNA with a limited number of genes and limited fold-changes. The results on SIRVs may not necessarily be generalisable to real biological RNA datasets. To address this, the authors could also run benchmarking on another biological RNA dataset by holding out a set of known transcripts from the annotation and assessing how many of them can be recovered in the output of each method.

The authors do not necessarily have to show that FLAIR2 outperforms all the existing isoform identification tools regarding overall performance. FLAIR2 includes transcript mapping, which could theoretically improve small or micro-exon detection. In addition, FLAIR/FLAIR2 could use the short reads to refine the splice junction, while the other benchmarked methods do not. The authors could potentially assess the performance of micro-exon and splice junction to highlight the benefits of using FLAIR2 for isoform detection. Besides, the potential of FLAIR2 to categorise haplotype-specific transcripts (HSTs) could still benefit the field - most other tools do not provide this option.

We thank the reviewer for accurately highlighing FLAIR’s features that distinguish them from other tools such as incorporating splice junctions from short-reads and identification of HSTs.

As mentioned, there is an extensive evaluation of FLAIR as part of LRGASP. We have now also included simulated data to demonstrate FLAIR2’s capability to identify HSTs with its assignment method. We simulated transcripts with a manually created, complex haplotype structure made to mimic A-to-I editing patterns. FLAIR2 was able to incorporate 15/15 variants correctly. We have also included an example with a small intron/deletion where FLAIR2’s improvements over its previous version are clearer. In the case of a very short intron, genomic alignments are less accurate than alignments to an annotated transcript, which FLAIR2 can now distinguish. These are both now supplemental figures just to showcase the new updates to FLAIR2.

2. In the section "Assessing FLAIR2 for haplotype-specific transcript detection", the authors identified 152 genes that contain HSTs and have shown some biological relevance of these genes. However, to highlight the novelty, it might be better to show that most of the genes can not be identified without using long reads. To address this, the authors could do a similar analysis that uses short reads and highlight that the number of HSTs or genes with HSTs are

significantly lower without long reads. Many haplotype specific analyses can be performed using short read data, but obviously fewer reads will be informative as they most will not include a relevant variant.

It has already been established that it is difficult to assemble full-length transcripts with short reads with accurate splice-junction chains (Steijger et al. Nature Methods 2013), and so transcripts found with short reads may not be comparable. Many existing HST callers rely on annotation (e.g. Sibbesen et al. Nature Methods 2023), so it is good to emphasize that with long-read sequencing methods, you are able to not only detect HSTs, but from unannotated transcripts.

3. In the section "Inosine detection in short and long reads", the authors contrast the significantly changed A-to-I events between samples with and without ADAR knockdown and claimed that long-read sequencing identifies transcripts that are missed from short-read sequencing methods, hence are advantageous for detecting certain novel A-to-I events. However, the overlap between the identification from long and short reads is very small, raising the concern that the additional events identified from the long reads might not be accurate. The authors might consider including more analysis to show that the "novel" events are not extensively false positives.

We manually reviewed novel events and additionally only considered events where we observed a decrease in editing upon ADAR knockdown. We now have included in the text a noted average decrease in Illumina coverage in these regions, as well as from manual review as shown in Fig 4c and 4d from poor coverage of the edit. Without additional validation, these novel positions can still be false positives despite our efforts to vet them. We have clarified in the text that these novel positions, while we try to implement filters, can still be false positives.

a) Could the authors please report the total number of A-to-I edits identified, out of which the significantly changed A-to-I events were identified? If the total number of A-to-I edits identified is relatively large, it might be helpful to do multiple testing corrections to prevent an extensive number of false discoveries. Otherwise, the authors should comment on why the false discoveries are not concerning in downstream analysis.

The total number of A-to-I events identified by only having those that are downregulated in the knockdown condition and also corroborated with REDIportal would be 62 events. We have tried multiple testing correction on the R2C2 data but with 4,000+ sites to test and generally lower coverage in R2C2 data, only 2 events are significant after correcting pvalues. We have added to the methods that no multiple testing was done for the nanopore A-to-Is identified (“After applying multiple testing corrections to these p-values, few events were significant so we only considered A-to-I discovery in the nanopore data as those with uncorrected p-values <0.05. We filtered for positions that had a minimum coverage of 10 in either condition and a change in percentage of edited reads after ADAR knockdown of 10% or more.”). Finally, we have also included in the discussion that these sites identified from R2C2 could be false positives. We try to demonstrate nanopore as a potentially useful tool for expanding inosine discovery, but is not the main

objective of FLAIR2, without additional validation. For inosines identified in R2C2 that are used in later analyses, we have provided examples of the data in the main and supplemental figures for readers to manually view the data.

b) It seems most of the A-to-I events, if significantly changed after ADAR knockout, are more expected to be down-regulated. Could the authors please report the number of significantly

up-regulated events to see whether an unexpected number is observed?

We have reported the number of significantly upregulated ADAR editing events from ADAR knockdown in Illumina (4 out of 413) and nanopore (1 out of 63) in the text. Fewer than 5% of significant events are upregulated in either short or long reads, which given our pvalue threshold of 0.05 should not indicate a concerning number of upregulated events.

c) Over half (68/131) of down-regulated A-to-I events are on novel A-to-I edit sites, which were not supported by short reads or in REDIportal. Given that these events were also considered when the authors identified "type II hyperediting" downstream, could the authors please explain why they are not identified by short reads if they are real? To address this the authors could include some statistics e.g., how many of the 68 events are from identified A-to-I sites (not necessarily downregulated) from short reads and their p-values. The idea is to verify the existence of any signal, even if it is weak, in short reads that could verify the novel events.

Regarding the 68 novel A-to-I sites from nanopore, as other reviewers have the same concern, we have softened the text describing these novel nanopore positions. We manually reviewed novel R2C2 events’ read level data in IGV and additionally only considered events where we observed a decrease in editing upon ADAR knockdown. We now have included in the text a noted average decrease in Illumina coverage in these regions, as well as from manual review as shown in Fig 4c and 4d from poor coverage of the edit. Without additional validation, these novel positions can still be false positives despite our efforts to vet them. We have clarified in the text that these novel positions, while we try to implement filters, can still be false positives.

4. Some presentation of the results are unclear and hard to understand. For instance, the numbers in the text seem not to be consistent with the results shown in Figure 2d. Figure 2d shows 413 significantly down-regulated A-to-I events, while the number is 312 at line 196. The authors should either revise the number or explain the inconsistency. Based on Figure 2d, the authors identified 63 significantly down-regulated A-to-I events from long reads overlapped with REDIportal. However, in lines 203-206, the authors stated that 63 significantly changed

long-read A-to-I events overlapped with REDIportal, and 62 were down-regulated.

We have corrected the Illumina discrepancy in the text now and it is consistent with Fig 2d. Now in the text: “We identified 413 A→G mismatches in the Illumina data that were significantly changed upon ADAR knockdown (Methods), with the majority (403) of these positions present in the REDIportal database [[49]](https://paperpile.com/c/UBnig6/At3U). Of the A-to-I events identified with short reads, 409 were downregulated in the knockdown conditions and 4 were upregulated.”

We have changed the figure legend for 2d Venn diagram from reporting the number of ‘significantly downregulated’ to the number of A-to-Is identified with ‘significant difference in

expression after ADAR downregulation’. In the text we have included further notes that of those with significant difference, how many are upregulated versus downregulated.

Minor comments

1. For reproducibility, it's better to provide the scripts used in all analyses, including the commands used when running FLAIR2. Particularly, it is not clear whether the short-read junctions were used in each FLAIR2 run.

We have added the flair collapse command to the methods. The short read junctions were used in the lung H1975 R2C2 FLAIR2 run where we had matched Illumina data, but not for the other runs. We have clarified this in the methods now too.

2. For SIRV analysis, it is not clear which SIRV annotation is provided. I believe there are different versions of annotation, including a complete annotation, an incomplete annotation and an over-annotated annotation.

We provided the complete annotation for the SIRVs (“C”), and have specified in the methods now.

3. In the FLAIR2 software GitHub page, it might be better to provide an example of data and code incorporating the longshot or PEPPER-margin-Deepvariant, and document the expected output.

We have added a page to describe the add-ons for FLAIR2 (https://flair.readthedocs.io/en/latest/flair2_functions.html),, including how to have FLAIR2 take in a file containing variant calls and also a file containing the FLAIR2 isoforms and read sequences to determine which isoforms contain which variants. It describes the output files (isoform structures, the subset of variants that were incorporated, and HST isoform sequences) and how to visualize HSTs (best to align the sequences and view in IGV to simultaneously see variant positions + isoform structures).

4. At line 161, when validating the 152 genes identified to contain HST bias, would it be possible to report the total number of known imprinting genes (perhaps after removing lowly expressed ones)? This might provide some idea of the sensitivity of the HST identification.

We tried to look for known imprinted genes by cross referencing [Santini et al. 2021’s](https://www.nature.com/articles/s41467-021-23510-4#MOESM4) list of imprinted genes in Supplementary Table 1 with our list of genes that contain multiple HSTs but few genes overlapped (9). We had only looked for HSTs in mouse with the definition that an transcript must have have increased representation of one haplotype compared to other isoforms for the same gene. This was only a subset of genes, as not all genes have multiple isoforms fitting this criterion.

5. In Table 3, it is not super clear why the number of size-selected reads is only for pool 3. It is also not clear what pool 3 is as it was not mentioned in the text. In the paragraph, starting at line 171, all the description was per-replicate instead of per-pool. It could be clearer if the authors also summarise the sequencing per replicate.

We have added much more text to the methods section to describe the pools more completely. Importantly, we clarify that each pool only contains 1 ADAR KD replicate and 1 CTRL KD replicate. We size selected one of the three pools (Pool 3) to increase the number of passes over the circularized molecule to increase consensus accuracy.

6. Line 202: could the authors please report the number of variants called by each variant caller and their overlaps to show the consistency of these variant callers?

We have added these numbers in – “Of the variants that overlapped with REDIportal, longshot identified 1,230 variants and PEPPER-Margin-DeepVariant identified 3,502. We combined all the variant calls from both tools for increased sensitivity, the union resulting in 4,020 putative

A-to-I events.” Default parameters of P-M-DV are more sensitive and there is an overlap of 712 variants that are putative A-to-Is (since we overlapped with REDIportal) called. Flair is not a variant caller so we did not evaluate which caller is more accurate and instead took the union and did other filtering to determine A-to-Is.

7. Line 215: It is not clear how much difference in the coverage between short reads and long reads. The authors could potentially report the average coverage.

Between Illumina and R2C2, the coverage difference is large and we have already implemented minimum coverage and significance filters for R2C2 inosine detection so we did not increase the coverage minimum there. For the editing sites detected only in R2C2, shown to be knocked down by A-to-I, and found in REDIportal and not Illumina, we have examined coverage differences and observed that those sites were more likely to be in repetitive regions and that Illumina read coverage was lower.

8. In the discussion, could the authors please comment on the potential usage of FLAIR2 on non-R2C2 data?

We have added this to the discussion. FLAIR2 was run on R2C2 for most instances of this paper. We comment in the discussion that FLAIR2 is still recommended for higher quality data to prevent false positives.

Reviewer #4: Tang et al. propose a haplotype specific transcript detection tool named FLAIR2, which is an extension to its previous version FLAIR. The major contribution of FLAIR2 is that it enables to integrate variant calls to discover haplotype specific transcripts, moreover, is able to detect the A-to-I editing site as well. The tool has its novelty, however, there is to-some-extent lack of systematic assessment in the manuscript so that the value of the tool is still hard to evaluate. I have some major concerns as following which should be addressed.

1. The sensitivity and precision of FLAIR2 were assessed with SIRVs, however, there is lack of in-depth analysis. For example, on the R2C2 data, FLAIR2 has higher precision but lower sensitivity than that of FLAMES. It is not shown that on what kind of cases that FLAIR2 failed to detect and make the false negatives? On the ONT 1D data, FLAIR2's sensitivity is obviously lower than that of Stringtie2, this also needs explanation. Moreover, FLAIR2 has a high improvement on precision than FLAIR, but also without a theoretical analysis and explanation in the manuscript. Such analysis on the method is critical to reflect the feature of FLAIR2 and to evaluate its contribution.

Besides performance metrics, we have included an example with a small intron/deletion where FLAIR2’s improvements over its previous version are clearer (Supplementary Figure 1). As for FLAIR2’s performance relative to other tools, this has been evaluated in LRGASP now which has benchmarked FLAIR. We now address this in the text. Please refer to the high-level response at the beginning of all our reviewer responses.

2. The assessment for haplotype-specific transcript detection is also a little over-simplified. Only 152 genes were analyzed, not on the whole genome/transcriptome level. Moreover, the authors only provide GO analysis and case studies. I think this is not enough, and a quantified assessment for the sensitivity and precision on the detection of HSTs on whole genome/transcriptome level is needed. It could be possible to employ some datasets with both of genomic and transcriptomic data to implement the benchmark and give the sensitivity and precision on whole transcriptome level.

The analysis was done at the whole genome level but only 152 genes returned HSTs using longshot to call a diploid haplotype and FLAIR2 for checking if the haplotypes were supported by a transcript’s supporting read sequences. We have dedicated more text to explaining how FLAIR’s assign_variants method works, which works by gathering the mismatches that are observed collectively in a read, and if observed 3 times or more, is output as an HST. We also now include more evaluation of FLAIR’s ability to detect HSTs using simulated data as there are no ground truth datasets for HST detection at a global level. We have clarified this in the text now.

3. The A-to-I editing site detection is another major contribution of FLAIR2. However, the assessment is also not very comprehensive. First-of-all, it does not show the precise results of the detection of A-to-I editing, such as the positions and distributions of editing sites. The authors give some indirect results, such as downregulation following ADAR1 knockdown and type II hyperediting, however, this is also not the assessment on whole transcriptome level. I think it could be helpful to ask FLAIR2 to output the A-to-I editing map, i.e., all the sites the tool detected, to assess its ability on whole transcriptome level.

We have now included all the detected positions of A-to-I edits from the Illumina and Nanopore data in Supplementary Data File 3 and indicate when these sites overlap or not. Supplementary Data File 1 contains the detected positions for Nanopore and also the counts of edited/unedited reads in the ADAR knockdown/control knockdown that were supplied to the Fisher’s test.

4. FLAIR2 detects transcripts from the read alignments in a two-step manner. In the second step, it detects novel transcripts based on the alignment between the unaligned long reads and reference genome. However, this is still a challenge since there are often complex alternative splicing events and very small exons. State-of-the-art read aligners usually produce noisy and inaccurate alignments around such sites. How does FLAIR2 handle those cases? Moreover, the alignments produced by various tools could be different from each other, is this also an issue affecting the results of FLAIR2?

It is true that novel transcript detection for FLAIR2 depends on the quality of the aligner. FLAIR2’s additional annotated transcript alignment step helps with challenging sites (microexons, subtle splicing changes, small introns as shown in what we have added as Supplementary Fig 1) so long as they are annotated. FLAIR2 does not have a way to deal with detection of novel microexons, which will be an area of future improvements. The best option is if you have short read sequencing and the splice junctions flanking the microexon have been recovered in short reads, these junctions can be supplied to minimap2 alignment, which accepts a junction.bed file to help guide the alignment. In the past, FLAIR has been tested on samples with splicing factor mutation and complex splicing. In these samples, we did not expect to detect much changes in alternative splicing from knocking down ADAR and should not affect FLAIR2 results.

5. FLAIR2 needs isoform annotations to implement the initial read alignment. However, it is not very clear about the details of the annotations used in the manuscript. This should clarify. Moreover, is the annotation critical to FLAIR2? That is, more comprehensive benchmark is still needed to assess the ability of FLAIR2 to handle novel transcripts.

We have added to the methods that the annotation we are using is Gencode v38. The annotation is critical in the sense that if only the annotation is supplied (and no short read information which would necessary for novel splice sites) then FLAIR will only create novel isoforms that are new combinations of known splice junctions. A comprehensive assessment of novel isoform detection was performed as part of the LRGASP study and FLAIR was determined to be one of the top performing tools for this purpose.

**Second round of review**

**Reviewer 1**

The comments have been addressed.

**Reviewer 2**

The authors have satisfactorily addressed my concerns in the revised manuscript and I do not have further comments.

**Reviewer 3**

I would like to express my appreciation for the authors' diligence and effort in addressing the comments and concerns. However, having carefully reviewed the revised version of the manuscript, I believe that there are some remaining concerns that need attention to meet the standards expected for publication.
It's good to see that the authors incorporated variant callers into the FLAIR pipeline, and I believe that FLAIR has the potential to serve as a valuable tool for detecting haplotype-specific transcript (HST) variation using long reads. In the manuscript, the authors have shown the potential of detecting HST, A-to-I editing that may not be found using short reads. However, the main concerns raised in our previous comment regarding the detailed performance of the tools do not seem to be fully addressed.

1. Regarding the previous comment: reviewer #3 major comment 1: In short, the concern was the lack of comprehensive benchmarking regarding isoform identification. It's fair that the authors addressed this by directing readers to the LRGASP paper, which has indeed shown a comprehensive evaluation regarding the isoform identification task. However, despite that the R2C2 data were available, the main sequencing protocol is R2C2 but the corresponding result of FLAIR was not included in the LRGASP paper. Showing detailed performance of FLAIR on R2C2 is crucial because high accuracy is essential for HST detection. Otherwise, the connection of the isoform identification section to the rest of the sections would be much weakened.
To address this the authors have added a sensitivity and specificity evaluation on R2C2 in this manuscript, but the analysis was much more simplified compared to the other sequencing protocols shown in the LRGASP paper. The results can be hard to generalise to real data because
1. the benchmarking is done on SIRV data with full annotation (i.e., a full set of ground truth) supplied, while in real data analysis, the annotation can often miss isoforms or contain redundant isoforms.
2. SIRV has its own limitations, as mentioned in the previous comment (reviewer #3 major comment 1c).

2. Another major concern (reviewer #3 major comment 3) is the lack of accuracy measurement of A-to-I event identification, which is one of the main contributions highlighted in the manuscript. The majority (68/131) of the identifications cannot be confirmed by REDIportal, which is derived from a large number of RNAseq studies, and more of them (104/131) cannot be confirmed by the matched short-read data. In the current writing, without measures of the accuracy of the 104/131 identified A-to-I events, the usibility of the output is limited.
More analysis can be done in this regard. In the manuscript, the authors showed some examples that some of the events can be due to the reduced short-read read depth, therefore, reduced statistical power. The authors could systematically check this category by checking the p-value of those long-read-only events in short reads (although they are not significant, they can provide some level of support to the event). The authors could also check whether there are long-read-only events that have a lot of short reads aligned, which can be likely false positives.

3. Similar accuracy concerns will also apply to the HST identification. The authors used simulated data for validation. However, the simulated reads are with great depth, and the location of simulated errors are almost random based on the badread algorithm. However, as R2C2 reads are derived from the consensus sequence, any persistant errors can be systematic errors (i.e., many reads from same transcript have similar errors at similar location), which is a major concern in the task of variant calling. The simulation presented in the manuscript was oversimplified and in a way that has minimised the difficulty of the variant calling task. Therefore, the presented simulation result has not provided strong support that the identified variants and HSTs were accurate.

4. (Reviewer #3 major comment 4) This is relatively minor, but the terms "down-regulated" and "significantly changed" are still mixed. For example, at line: "We found that approximately half (79/131) of the significantly downregulated inosines were considered type I hyperedited in the control knockdown data." I believe the number 131 refers to the significantly changed sites instead of significantly down-regulated inosines based on update figure. Also, it's still not clear whether there is any up-regulated A-to-I site within the 68 novel events not supported by short reads or REDIportal.

**Authors’ response to reviewers**

We are happy to see that all concerns of Reviewers #1 and #2 were addressed in our last version and we now address the additional concerns raised by Reviewer #3, below.

Reviewer #3: I would like to express my appreciation for the authors' diligence and effort in addressing the comments and concerns. However, having carefully reviewed the revised version of the manuscript, I believe that there are some remaining concerns that need attention to meet the standards expected for publication.
It's good to see that the authors incorporated variant callers into the FLAIR pipeline, and I believe that FLAIR has the potential to serve as a valuable tool for detecting haplotype-specific transcript (HST) variation using long reads. In the manuscript, the authors have shown the potential of detecting HST, A-to-I editing that may not be found using short reads. However, the main concerns raised in our previous comment regarding the detailed performance of the tools do not seem to be fully addressed.

1. Regarding the previous comment: reviewer #3 major comment 1: In short, the concern was the lack of comprehensive benchmarking regarding isoform identification. It's fair that the authors addressed this by directing readers to the LRGASP paper, which has indeed shown a comprehensive evaluation regarding the isoform identification task. However, despite that the R2C2 data were available, the main sequencing protocol is R2C2 but the corresponding result of FLAIR was not included in the LRGASP paper. Showing detailed performance of FLAIR on R2C2 is crucial because high accuracy is essential for HST detection. Otherwise, the connection of the isoform identification section to the rest of the sections would be much weakened.
To address this the authors have added a sensitivity and specificity evaluation on R2C2 in this manuscript, but the analysis was much more simplified compared to the other sequencing protocols shown in the LRGASP paper. The results can be hard to generalise to real data because
1. the benchmarking is done on SIRV data with full annotation (i.e., a full set of ground truth) supplied, while in real data analysis, the annotation can often miss isoforms or contain redundant isoforms.
2. SIRV has its own limitations, as mentioned in the previous comment (reviewer #3 major comment 1c).

We recognize the importance of benchmarking on non-synthetic, R2C2 data. The LRGASP data thus far had shown comparable or better performance of isoform detection by FLAIR2 in ONT and PacBio in synthetic and real datasets. The higher accuracy of PacBio reads is similar to R2C2 ONT, but a direct comparison of FLAIR2 to other tools with R2C2 data would bolster any claims to improvement of FLAIR2 over the original FLAIR for this paper in particular, which mainly analyzes R2C2 data. Thus, in this resubmission we have now included additional benchmarking with FLAIR2 isoform detection on the LRGASP WTC-11 R2C2 datasets. The results are now updated in Supplementary Table 2.

We have included results for two additional LRGASP benchmarks with R2C2 data. The first is an evaluation againts GENCODE v39, which was released after the close of challenges. GENCODE human v38 was available at the time of the LRGASP data release. Therefore, this benchmark evaluates performance when there are missing isoforms from the annotation. The second benchmark is from a set of 50 loci that were manually annotated by GENCODE annotators using the real LRGASP data from each library and contains curated GENCODE-annotated transcripts as well as novel transcripts that are not present in the GENCODE reference.

For the benchmark against GENCODE v39, we summarize the performance based on transcript support with orthogonal data. For the human WTC11 cell line, LRGASP also produced orthogonal CAGE-seq data (sequencing of 5’ end of transcripts) and Quant-seq data (sequencing of the 3’ end of transcripts). Splice junction coverage from short-read Illumina sequencing was also used for evaluation. FLAIR is the only tool that had the top 3 performance using all metrics including the percentage of annotated transcripts with full orthogonal support (%SRTM: 5’ end, 3’ end, and splice junction support) and percentage of novel transcripts with full orthogonal support (%SNTM).

When using the GENCODE manual annotation as a benchmark, FLAIR had the best performance for sensitivity to novel transcripts with the second best performance for precision of novel transcripts. From these results, we conclude that even on real datasets, FLAIR2 is a top-performing tool and can identify novel isoforms with good sensitivity and precision.

Relevant text has been added to the manuscript:
“Additionally, we assessed FLAIR2 using the WTC-11 R2C2 data from LRGASP with benchmarks using orthogonal data support and a manual annotation performed by GENCODE. FLAIR is the only tool that had the top 3 performance using all metrics including the percentage of annotated transcripts with full orthogonal support (%SRTM: 5’ end CAGE-seq, 3’ end Quant-seq, and short-read splice junction support) and percentage of novel transcripts with full orthogonal support (%SNTM) (Supplementary Table 2). Using the GENCODE manual annotation as a benchmark, all tools had a weaker performance for novel transcript detection; however, FLAIR had the best sensitivity and 2nd best precision for detecting novel transcripts (Supplementary Table 2). Overall, FLAIR2 has improved its transcript detection approach over the previous version and is one of the top performing tools for both annotated and novel transcript isoform detection using a variety of library preparation methods and sequencing approaches.”


2. Another major concern (reviewer #3 major comment 3) is the lack of accuracy measurement of A-to-I event identification, which is one of the main contributions highlighted in the manuscript. The majority (68/131) of the identifications cannot be confirmed by REDIportal, which is derived from a large number of RNAseq studies, and more of them (104/131) cannot be confirmed by the matched short-read data. In the current writing, without measures of the accuracy of the 104/131 identified A-to-I events, the usibility of the output is limited.
More analysis can be done in this regard. In the manuscript, the authors showed some examples that some of the events can be due to the reduced short-read read depth, therefore, reduced statistical power. The authors could systematically check this category by checking the p-value of those long-read-only events in short reads (although they are not significant, they can provide some level of support to the event). The authors could also check whether there are long-read-only events that have a lot of short reads aligned, which can be likely false positives.

We have checked whether the 68 nanopore-only events were recovered by at least one Illumina ctrl kd replicate, which was not enough to be a called event in the short read data but to see if any evidence of the edit existed in the Illumina data and was not a consistent error in the R2C2 data. 53 of the nanopore-only sites were corroborated with Illumina in this way. Manual inspection of all 68 sites revealed that many sites had less coverage in the Illumina data (in agreement with the previously calculated coverage comparison of these sites that were missed by short reads) but still supported the edit, and the magnitude of knockdown was not a strong as in the nanopore data due to variation in sampling. The decrease in coverage and/or lack of consistency across the Illumina replicates would result in that site being dropped from the DRIMSeq significance testing or that site not being called significantly different between ADAR kd/control kd.

For nanopore-only sites that could not be corroborated by any Illumina reads, we investigated whether these were due to mapping difficulties, such as in the case of MREG Fig 4C. Previous literature has shown that A-to-I editing can affect alignment rates; to check this, we created an edited version of the hg38 genome reference with the nanopore-only A-to-I edits and aligned the unmapped Illumina reads. From this analysis, we found that we were able to rescue some previously unmapped edited Illumina reads for 28 sites.

However, not all of these sites can be explained in the above ways regarding coverage/alignment issues. There are a minority of sites found with nanopore and, despite many Illumina reads aligned at that position, have no support in Illumina. Reasons for false positives could be homopolymers, a lower allele expression, or possibly systematic errors in nanopore. We have chosen to still report all the significantly different inosines found with nanopore but emphasize our confidence in those that have some support in Illumina as we do not have orthogonal validation for our nanopore-only putative inosines.

We have also been more careful in the text to refer to the novel inosines discovered only with R2C2 as ‘putative’.

Places in the main text that address this issue now:
Results: “On detecting multiple edits in short-read RNA-Seq, if the edits are too distant, or if a read contains many mismatches on account of A-to-I hyperediting (type II), reads with multiple edits may not align to the genome and evade detection [53]”
Results: “We also noted that 60 of the 68 putative A-to-I events that were identified as significantly differentially edited in only the R2C2 data were downregulated in the knockdown condition. Even though these events were nanopore-specific, we observed evidence that the Illumina data may be lacking coverage at these sites. For example, some of the nanopore-only events in the Illumina replicates received insufficient aligned coverage or did not have enough edited reads to pass a significance threshold (Fig 3, Supplementary Fig 3). We computed the coverage of inosines that were called in Illumina data and the coverage of those that were missed by Illumina but found to be significantly knocked down in the R2C2 data and validated with REDIportal, and the latter positions had significantly lower coverage (mean coverage difference of 31.3, Mann-Whitney U p-val 0.0176). Of the 68 significant putative inosines in nanopore, 53 had reads in at least one control knockdown Illumina sample supporting the edit. To help improve alignments, we aligned unmapped Illumina reads to an edited version of the hg38 reference with specific nucleotides replaced with the nanopore-identified differentially edited sites and found that we were able to increase coverage for 28 sites. In conclusion, while the coverage of short read data will typically surpass that of long reads lending to an increase in the number of inosines detected, long reads could be advantageous for detecting certain A-to-I events.”
Discussion: “We were able to discover putative novel type I and type II hyperediting (Fig 2e and 3a), sites that are coordinated with each other (Fig 4b), and sites that may disrupt splicing (Fig 4c,d)...We observed a general decrease in Illumina coverage of the novel nanopore sites that are missed in short read data, indicating potential alignment challenges for short reads with many A-to-I mismatches such that only unedited reads align. While such sites may not have been identified by Illumina due to coverage issues, many sites could still be corroborated with short read realignments, increasing our confidence in them. While we considered novel, putative inosines detected only in R2C2 data as those that we observed significant downregulation of upon ADAR knowndown, additional validation is necessary to distinguish true novel inosines from any false positives arising from homopolymer or systematic errors.“


3. Similar accuracy concerns will also apply to the HST identification. The authors used simulated data for validation. However, the simulated reads are with great depth, and the location of simulated errors are almost random based on the badread algorithm. However, as R2C2 reads are derived from the consensus sequence, any persistant errors can be systematic errors (i.e., many reads from same transcript have similar errors at similar location), which is a major concern in the task of variant calling. The simulation presented in the manuscript was oversimplified and in a way that has minimised the difficulty of the variant calling task. Therefore, the presented simulation result has not provided strong support that the identified variants and HSTs were accurate.

In the case of the simulation, we only provided known, simulated variant calls to flair-assign, removing any variant calling variability from the assessment since the goal of the simulation was to test the integration of known variants into isoform models. We have now emphasized in the text and updated Figure 1A that FLAIR2 does not perform variant calling itself, and that FLAIR2 is connecting variants to specific transcripts. Besides the simulation, we also have testing of the FLAIR2 HST identification with the hybrid mouse ES haplotype identification, although this is real data without a gold standard. For the HST analysis in the mouse data, like with the simulation, our objective is to demonstrate that specific variants can be linked to specific transcripts with FLAIR; these variants may need to be further vetted so we have noted this in the paper as well now.

For this paper, we focused very specifically on variant calling in long reads to identify A-to-G SNPs, honing in on SNPs that were changed upon ADAR knockdown. While it is true that nanopore reads (R2C2 or other) can suffer from homopolymers, indels, and systematic errors that make variant calling challenging even with regular improvements to the technology, we have determined that the ability to variant call from the long read transcriptome out of the scope of this paper because of this paper’s focus on inosine characterization. Variant calling with nanopore reads can be challenging, and we mention looking at both longshot and deepvariant as tools that are tackling this.

In the text:
Results: “We provide an example of complex multiple haplotype calling, where, given variant calls with simulated nanopore data with 99% accuracy and sufficient coverage of each haplotype, FLAIR2 incorporates 15/15 variants correctly (Supplementary Fig 2).”
Discussion: “It should be noted that FLAIR2 itself is not a variant caller, but can integrate variants called from other tools or vetted by other sources to assemble into HSTs.”
In figures:
Updated Figure 1A indicates that variant calls using aligned cDNA/RNA bams will originate from another tool, i.e. longshot, which has been developed for diploid, error-prone reads.


4. (Reviewer #3 major comment 4) This is relatively minor, but the terms "down-regulated" and "significantly changed" are still mixed. For example, at line: "We found that approximately half (79/131) of the significantly downregulated inosines were considered type I hyperedited in the control knockdown data." I believe the number 131 refers to the significantly changed sites instead of significantly down-regulated inosines based on update figure. Also, it's still not clear whether there is any up-regulated A-to-I site within the 68 novel events not supported by short reads or REDIportal.

You have correctly pointed out a discrepancy and keeping ‘down-regulated’ and ‘significantly changed’ is an important distinction. We have modified the text from significantly downregulated to significantly changed for the 131 nanopore A-to-Is that were significant. And for the 68 sites that are significantly changed in only R2C2 and not short reads or REDIportal, 60 are downregulated; we have noted this in the text now as well.

**Third round of review**

**Reviewer 3**

We thank the authors for their revisions and do not have any further comments.
